# Quinoline‐Conjugated Ruthenacarboranes: Toward Hybrid Drugs with a Dual Mode of Action

**DOI:** 10.1002/cmdc.201900349

**Published:** 2019-11-19

**Authors:** Marta Gozzi, Blagoje Murganic, Dijana Drača, John Popp, Peter Coburger, Danijela Maksimović‐Ivanić, Sanja Mijatović, Evamarie Hey‐Hawkins

**Affiliations:** ^1^ Institute of Inorganic Chemistry Leipzig University Johannisallee 29 04103 Leipzig Germany; ^2^ National Institute of Republic of Serbia Department of Immunology Institute for Biological Research “Siniša Stanković” University of Belgrade Bul. despota Stefana 142 11060 Belgrade Serbia

**Keywords:** ruthenacarborane, autophagy, quinoline, self-assembly, glioblastoma

## Abstract

The role of autophagy in cancer is often complex, ranging from tumor‐promoting to ‐suppressing effects. In this study, two novel hybrid molecules were designed, containing a ruthenacarborane fragment conjugated with a known modulator of autophagy, namely a quinoline derivative. The complex *closo*‐[3‐(*η* ^6^‐*p*‐cymene)‐1‐(quinolin‐8‐yl‐acetate)‐3,1,2‐RuC_2_B_9_H_10_] (**4**) showed a dual mode of action against the LN229 (human glioblastoma) cell line, where it inhibited tumor‐promoting autophagy, and strongly inhibited cell proliferation, *de facto* blocking cellular division. These results, together with the tendency to spontaneously form nanoparticles in aqueous solution, make complex **4** a very promising drug candidate for further studies *in vivo*, for the treatment of autophagy‐prone glioblastomas.

## Graphical abstract

**Blasting glioblastoma**: Autophagy has become a very attractive target for the design of novel chemotherapeutic agents. Herein we describe the synthesis, aqueous solution properties, and *in vitro* biological evaluation of ruthenacarborane complexes conjugated with quinoline derivatives that act as autophagy modulators against both human breast adenocarcinoma (MCF‐7) and human glioblastoma (LN229) cell lines.

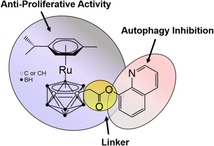

## 1 Introduction

Polyhedral molecular boron‐carbon clusters (carboranes) of type *closo*‐C_2_B_10_H_12_, *nido*‐[C_2_B_9_H_12_]^−^, *nido*‐[C_2_B_9_H_11_]^2−^ and their metal complexes, are already well‐established scaffolds in the medicinal inorganic chemistry.^1^ By far the most extensively studied application of carboranes in medicine is their use as high‐boron carriers for boron neutron capture therapy (BNCT),^2^ followed by pharmacophores in drug design3a, 3b and radio‐imaging agents.^4^, ^5^ Regardless the particular type of cluster, carborane‐containing molecules and complexes have been intensively studied for targeting cells and tissues within the central nervous system (CNS),6a–6d because they are able to efficiently cross the blood‐brain‐barrier (BBB, the “brain keeper”), thanks to the presence of hydridic B−H bonds, which make the cluster highly hydrophobic.^7^

During our investigations on the medicinal chemistry of ruthena‐ and molybdacarboranes,^8^, ^9^, ^10^ we showed that these complexes spontaneously self‐assemble in aqueous solutions, forming nanoparticles.^10^ Using bovine serum albumin (BSA) as nanocarrier system allows to control the size of the nanoparticles to ca. 100 nm,^9^ which falls within the optimal size range for application of nanoparticle‐based drug delivery technologies, in terms of cellular uptake and clearance pathways.^11^ Thus, this spontaneous property of the metallacarborane fragment is well‐suited for designing novel chemotherapeutic agents to target those types of tumor, where nano‐sized chemotherapeutics might provide superior efficacy compared to non‐nano‐sized drug formulations, due to the characteristics of the tumor itself, as proposed recently, for example, for brain tumors of the glioblastoma type (GBM).^12^ The latter are in fact tumors characterized by extensive and irregular vasculature, which makes them optimal targets for drug delivery systems that can exploit the “enhanced permeability and retention effect” (EPR) of cancer cells with respect to healthy cells, thus promoting tumor‐selectivity of the treatment. The big advantage in using metallacarboranes is that there is no need for engineering the nanoparticles, they are spontaneously formed. In this work, the metallacarborane unit should act, therefore, as pharmacophore and delivery system.

**Figure 1:**
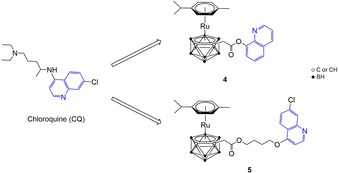
Molecular structure of quinoline‐conjugated ruthenacarboranes **4** and **5**, synthesized in this work (right). The lead organic structure (chloroquine, CQ) is shown on the left. The 1‐aza‐naphthalene heterocycle is highlighted in blue.

We chose to combine an [(*η* ^6^‐arene)Ru]^2+^‐carborane unit with a 1‐aza‐naphtalene‐based organic residue, commonly known as quinoline (Figure 1). Quinoline derivatives possess a plethora of biological activities,13a, 13b which have prompted the development of numerous drugs, ranging from antiparasitic (e. g. chloroquine and its ferrocene analogue ferroquine), to antiviral (e. g. saquinavir), antibacterial (e. g. ciprofloxacin), anti‐inflammatory (e. g. quinoline alkaloids), antioxidant (e. g. quinoline glycoconjugates), antineoplastic (e. g. irinotecan), and many others.14a–14c The specific type and position of substituents on the heterocycle influence the physicochemical and pharmacologic properties of the quinoline‐containing molecules, and thus modulate the activity of the drug, as reported for example by Natarajan et al. in a structure‐activity relationship (SAR) study on the influence of side chain length and quinolyl nitrogen *p*K_b_ on the antimalarial activity of chloroquine.^15^ Combinations of a ruthenium(II)‐arene fragment with chloroquine (CQ) or 8‐hydroxyquinoline (8‐HQ) derivatives have been reported by many in the literature (Gobec et al., Mitrović et al., Kubanik et al., Glans et al., Movassaghi et al., Martinez et al.), showing modulation of the biological activity with respect to the two scaffolds alone, for potential applications as chemotherapeutic agents in different types of tumors (e. g. leukemia, colorectal, lung and cervical cancers),16a–16c, 17, 18 and as antiparasitic agents.19a–19c

For the present work, we were particularly interested in the activity of substituted quinolines as modulators of macroautophagic processes,^20^, ^21^, ^22^ often simply called autophagy. The latter is an ensemble of physiologic catabolic processes,^23^, ^24^ which break cytosolic proteins and organelles down to their building blocks, e. g. amino acids, in response to a plethora of stressors including starvation, oxidative stress and pathogens, and act also as a quality control system, to ensure homeostatic functions and organelle turnover. Strong evidence has been collected in the last 20 years which supports a dual role of autophagy in cancer, either in tumor suppression or in tumor promotion, depending on the specific cancer type and the stage.^25^, ^26^ Thus, the complex plethora of phenomena which underlie the cellular autophagic response, has become today a very attractive *biochemical* target for anti‐cancer therapies, although the fundamental genetic mutations and biochemical processes are, for many cases, not fully elucidated:^26^ its modulation was shown to promote (re)sensitization of cancer cells to the applied treatment, either cytotoxic agents (chemotherapy) or radiation therapy, for cases of pancreas, breast and prostate cancers, and gliomas.27a–27d

CQ and its derivative hydroxychloroquine (HCQ) are known inhibitors of autophagy,^20^, ^21^ used in the clinics to treat GBM tumors, typically in combinatorial therapies together with the alkylating agent temozolomide (TMZ), and contribute, in some cases, to extend survival prognoses in patients affected by these aggressive, autophagy‐prone tumors.^28^ The *N*,*N*‐(8‐hydroxyquinoline)methyl‐substituted benzylamine JLK1486 has been reported to inhibit cellular proliferation in B16F10 skin melanoma cells, via induction of cytodestructive autophagy.^29^ The styrylquinoline LV‐320 was found to block the autophagic flux, and thus impair cellular viability, in several breast cancer cell lines, in a dose‐dependent manner.^30^

Here, we aimed at studying the modulation of the biological activity of a ruthenacarborane fragment upon conjugation with a quinoline residue, specifically investigating the modulation of the cellular autophagic response after treatment with the ruthenacarborane alone, or with its quinoline conjugates. As linker, a carboxylic acid ester was chosen, and its stability toward hydrolysis at physiologic pH (7.4) was investigated. The aqueous self‐ and co‐assembly behavior of the ruthenacarborane ester **4** and the corresponding free acid (**3**), with and without BSA, was studied via Nanoparticle Tracking Analysis (NTA). The complexes were screened against a panel of cancer cell lines, including human breast adenocarcinoma (MCF‐7) and human glioblastoma (LN229) cells. Their mechanism of action was further investigated via flow cytometry, fluorescence microscopy and wound healing assay, and the presence of autophagy marker LC3B (microtubule‐associated light chain protein 3B) was evaluated via western blot analysis.

## 2 Results and Discussion

### 2.1 Synthesis and Characterization

**Scheme 1:**
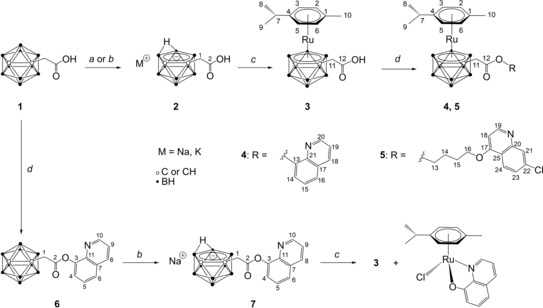
Synthetic approaches to target ruthenacarborane complexes **4** and **5**, from *closo*‐carborane derivative **1**. (*a*) i) KOH (3.5 eq.), EtOH, reflux, 21 h; ii) HCl_aq_. (*b*) NaF (5.0 eq.), EtOH/H_2_O 3 : 2 (v/v), 90 °C, 18 h. (*c*) i) TlOEt (3.0 eq.), THF, −35 °C to rt, 2 h; ii) [{(*η* ^6^‐*p*‐cymene)RuCl(μ‐Cl)}_2_] (0.5 eq.), CH_2_Cl_2(deg.)_, −65 °C to rt, 18 h; iii) HCl_aq_. (*d*) Boc_2_O (1.5 eq.), 8‐hydroxyquinoline (for **4**, **6**) or [(7‐chloroquinolin‐4‐yl)oxy]butan‐1‐ol (for **5**) (1.25 eq.), NaNH_2_ (1.25 eq.), CH_2_Cl_2_/pyridine 10 : 1 (v/v), −35 °C to rt, 19 h.

Ruthenacarborane complex **3** (i. e. free acid) was synthesized in two steps from *closo*‐1‐(CH_2_COOH)‐1,2‐C_2_B_10_H_11_ (**1**) (Scheme 1). A methylene spacer between the cluster and the carboxylic acid group was preferred over no spacer, i. e. the formic acid‐substituted *closo*‐1‐(COOH)‐1,2‐C_2_B_10_H_11_, due to the well‐known tendency of an electron‐withdrawing carboxylic acid group to spontaneously undergo decarboxylation, when attached directly to the cluster, which could also, prospectively, impair biological stability of the complex. Deboronation of **1** proceeded smoothly in KOH/EtOH at reflux overnight, or, alternatively, with excess NaF in EtOH/H_2_O (3 : 2 (v/v)) at 90 °C, in an analogous way as described by El‐Zaria et al. for guanidine‐substituted *closo*‐*ortho*‐carboranes.^31^ The same approach was successfully applied for the deboronation of the quinolin‐8‐yl ester **6**, which gave straightforward access to *nido*‐carborane(−1) **7** as its sodium salt, in 64 % yield. **3** was obtained via salt‐metathesis from the dithallium salt of **2** and [{(*η* ^6^‐*p*‐cymene)RuCl(*μ*‐Cl)}_2_], which is a standard approach to ruthenacarboranes,32a, 32b in low yield (36 %) after tedious separation via column chromatography. Activation of the carboxylic group of **3** with di‐*tert*‐butyl dicarbonate (Boc_2_O), in CH_2_Cl_2_/pyridine, followed by reaction with sodium quinolin‐8‐olate (or [(7‐chloroquinolin‐4‐yl)oxy]butanol‐1‐ate), yielded ruthenacarborane esters **4** and **5**, in 18 % and 21 % yield, respectively. 8‐hydroxyquinoline (8‐HQ) was chosen due to the plethora of studies which combine a ruthenium(II)‐arene fragment with 8‐HQ,^17^, ^18^, ^33^ and [(7‐chloroquinolin‐4‐yl)oxy]butanol because of its structural similarity to CQ, but still suitable for the synthesis of an ester bond. The low yields of isolated final products can be attributed, at least partially, to the instability of the carborane‐bound ester bonds on chromatography columns (see also ester **6** (19 %)), as we have frequently observed in our group, for both ester and amide bonds (see for example ref. [34]). Interestingly, the carboxylic group of **1** and **3**, which are structurally both *closo* species, could be activated under analogous conditions (Scheme 1), to form an ester bond, with both an aromatic (**4**, **6**) and an alkyl (**5**) alcoholate. Reaction of the dithallium salt of **7** with [{(*η* ^6^‐*p*‐cymene)RuCl(*μ*‐Cl)}_2_] failed to give access to the desired ruthenacarborane ester, but yielded instead **3**, i. e. the free acid, and a second carborane‐free ruthenium(II)‐arene complex, identified via ^1^H NMR spectroscopy as chlorido(8‐quinolinato‐κ^2^ *N*,*O*)(*η* ^6^‐*p*‐cymene)ruthenium(II) (Figure S1, SI), already reported in the literature.^18^

^1^H and ^11^B NMR (CDCl_3_) spectroscopic analysis of the deprotonation of **7** with thallium ethanolate (TlOEt, step *c* in Scheme 1) confirmed quantitative conversion to the dithallium salt without cleavage of the ester bond, whereas no NMR spectrum of the dithallium salt of **2** (i. e. free acid) could be measured in CDCl_3_, due to its very low solubility (Figures S2 and S3, SI). Changing the base from TlOEt to the bulky lithium bis(trimethylsilyl)amide (LiHMDS) or changing the stationary phase from silica gel to neutral alumina for the chromatographic separation of the crude product yielded again only **3**. So, it is not a nucleophilic base, nor the purification method, which completely cleaves the ester bond, but probably cleavage already occurs during the complexation reaction itself, when the Ru^2+^ center can be coordinated by the nitrogen and oxygen atoms of the quinoline ring, besides the C_2_B_3_ face of the dicarbollide cluster.

**Figure 2:**
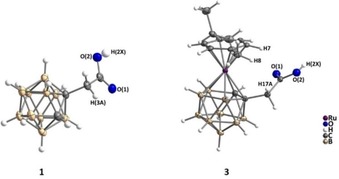
Molecular structures of **1** (left) and **3** (right). Thermal ellipsoids at 50 % probability level. Labelling of selected atoms is given.

The unsymmetrical substitution of the cluster carbon atoms introduces chirality to the molecule, or metal complex, as evidenced in the ^1^H NMR spectra of **2**–**5** and **7**. The two α‐methylene protons (*H* ^1^ for **2** and **7**, *H* ^11^ for **3**–**5**) are magnetically not equivalent, due to the presence of the C_2_B_3_ chiral plane, and thus appear as two doublets, with geminal coupling constants of 16–17 Hz (Figure S4, SI). All compounds were synthesized as racemic mixtures and used as such. ^1^H NMR signals for the ruthenacarborane unit are almost identical in **3**, **4** and **5**, in CDCl_3_. The same holds for the ^11^B NMR spectra, which shows that modifications of the cluster‐bound carboxylic acid group have little to no influence on the electron distribution throughout the cluster. Crystallographic study of **1** and **3** showed that the two fragments [C_2_B_10_H_11_] and [RuC_2_B_9_H_10_] are isolobal species, with **3** derived from formal replacement of the B(3)−H group of **1** with the [Ru(*η* ^6^‐*p*‐cymene)]^2+^ fragment, which results in a distorted *closo* structure (Figure 2). The *p*‐cymene ligand in **3** is bent toward the boron atoms of the C_2_B_3_ face, which results in higher deviation from coplanarity of the aromatic C_6_ and the B_5_H_5_ ring (6.84(7)°, Table 1), and a higher tilt angle between C_6_ and the C_2_B_3_ ring (8.19(7)°), with respect to the corresponding unsubstituted complex [3‐(*η* ^6^‐*p*‐cymene)‐3,1,2‐RuC_2_B_9_H_11_] (5.11(9)° and 6.25(7)°).^8^ This is partially due to the stronger *trans* influence of the boron atoms compared to carbon,^35^ but also to electronic repulsion between O(1)/O(2) (carboxy group) and the π system of the arene ligand, rather than to steric crowding, as observed instead by Welch and co‐workers for ether‐substituted ruthenacarboranes^36^ (in the case of **3**, the lowest intramolecular H⋅⋅⋅H distances (H(17 A)⋅⋅⋅H(7) 2.668(1) Å; H(17 A)⋅⋅⋅H(8) 2.762(1) Å) are larger than the sum of their van der Waals radii).

**Table 1:** Selected bond lengths (Å) and angles (°) for **1** and **3**.

	**1**	**3**
C−C(cluster)	1.651(5)	1.665(3)
B−B^[a]^	1.776(6)	1.779(5)
B−C(cluster)^[a]^	1.710(5)	1.720(4)
Ru−C(C_2_B_3_ face)^[a]^	–	2.183(2)
Ru−B(C_2_B_3_ face)^[a]^	–	2.205(3)
B(3)−C(C_2_B_3_ face)^[a]^	1.705(5)	–
B(3)−B(C_2_B_3_ face)^[a]^	1.754(6)	–
Ru−Ctd1^[b]^	–	1.725(3)
Ru−Ctd2^[b]^	–	1.614(3)
Ru−C(*p*‐cymene)^[a]^	–	2.231(3)
H(2X)⋅⋅⋅O(1)	1.698(6)	1.865(3)
H(3 A)⋅⋅⋅O(1)	2.516(1)	–
Deviation from coplanarity^[c]^	–	6.84(7)

[a] Average value. [b] Ctd1=centroid of the C_6_H_4_ ring of the *p*‐cymene ligand. Ctd2=centroid of the C_2_B_3_ face of the dicarbollide ligand. [c] Deviation from coplanarity of the arene (*p*‐cymene) and dicarbollide ligands was measured between the least‐squares plane formed by the C_6_H_4_ ring of the arene ligand and the least‐squares plane formed by the lower boron belt (B_5_H_5_) of the cluster, as reported previously.^24^

The packing is stabilized by intermolecular hydrogen bonds (H(2X)⋅⋅⋅O(1) for **1** and **3**, H(3 A)⋅⋅⋅O(1) for **1**). One molecule of **1** forms two hydrogen bonds with two other molecules, resulting in a “polymer”‐like 3D network, whereas one molecule of **3** forms a centrosymmetric dimer, which is often observed for carboxylic acids (Figure S8, SI).^37^

Thus, of the two synthetic approaches tested for the synthesis of ruthenacarborane esters, the activation of the free carboxylic group of complex **3** (step *d* in Scheme 1) with Boc_2_O in CH_2_Cl_2_/pyridine, followed by reaction with an aryl or alkyl alcoholate, gave straightforward access to the desired quinoline esters **4** and **5**. That one can make use of one unique building block, such as the carboxylic acid **3**, to attach different types of substituents (e. g. aryl or alkyl groups), represents an enormous advantage in the synthetic chemistry of metallacarboranes, and is in fact a broadly used approach, both for full‐ and half‐sandwich metallacarboranes, alike.38a–38c To the best of our knowledge, this is the first report on the use of a carboxylic acid‐functionalized ruthenacarborane as building block for the synthesis of esters, designed for triggering a specific biological response. The successful activation of the carboxylic acid group in complex **3** paves the way for the synthesis of a multitude of rationally designed complexes, which might also incorporate, for example, biomolecules, such as specific peptides for target‐vector recognition mechanisms.

### 2.2 Stability Studies

Stock solutions of sparingly water‐soluble compounds for *in vitro* cell cultures are usually prepared in DMSO, ethanol or methanol, and stored frozen over months, provided that the compound shows the necessary chemical stability (no or minimal ligand dissociation, in the case of metal complexes). **3** and **4** were found to be stable in water‐containing DMSO‐d_6_ solution, in air, for over a month. No changes were detected in either ^1^H or ^11^B{^1^H} NMR spectra (Figures S5 and S6, SI). **5** showed no changes in the ^11^B{^1^H} NMR spectrum, but a second set of signals for the [(7‐chloroquinolin‐4‐yl)oxy]butanol‐1‐yl group appeared in the ^1^H NMR spectrum, right after dissolution in DMSO‐d_6_, and remained constant in shift and intensity over one month (Figure S7, SI). These signals cannot be attributed to free 4‐{(7‐chloroquinolin‐4‐yl)oxy}butanol‐1‐ate, but rather indicate the presence of a second tautomer (ca. 5 % at 25 °C), which is common in polar solvents for quinoline‐containing groups.^39^

Following our recent investigations on the self‐assembly behavior of ruthenacarborane complexes in aqueous solutions at physiologic pH,^9^, ^10^ the behavior of free acid **3** and ester **4** in aqueous solution was studied via UV‐vis spectroscopy and Nanoparticle Tracking Analysis (NTA) in phosphate‐buffered saline (PBS)/DMSO mixtures (pH 7.4). UV‐vis spectroscopy is a useful tool to follow the ester hydrolysis over time, when a UV‐(vis)‐active chromophore is present (in the case of **4**, the quinoline ring). Experiments were carried out at room temperature (23 and 25 °C, for UV‐vis and NTA, respectively), to simulate the conditions of the working solutions used for cell cultures, and at 37 °C (UV‐vis), to simulate the conditions of incubation of cells.

**Figure 3:**
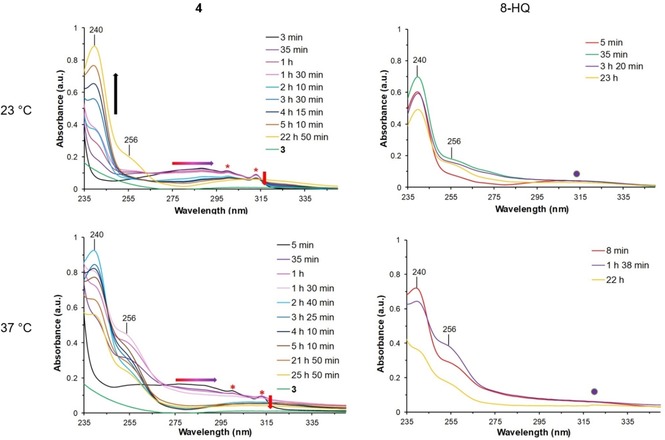
Time‐resolved UV‐vis spectra of **4** (left) and 8‐HQ (right) in PBS solution, at 23 °C (top) and 37 °C (bottom). **3** in PBS solution is also shown, as reference. Vol% DMSO is 1 % for all samples. λ_max_ 240 and 256 nm (characteristic of the quinoline group) are marked in all spectra. For **4** at 23 °C (top left), the black arrow indicates the time evolution of the band at λ_240_. Red indicates bands characteristic of **4** only, purple of 8‐HQ only. * highlights the two sharp transitions for **4**. For **4**, the red arrow indicates the time‐evolution (decrease) of the sharp band at λ_314_, the red‐to‐purple arrow indicates the red shift of the broad band, from 250–320 nm (characteristic of **4**, red) to 290–340 nm (characteristic of 8‐HQ, purple).

**4** shows one broad absorption band in the range 250–320 nm, right after dissolution in PBS/DMSO (black curve, Figure 3, left), which is blue‐shifted with respect to the corresponding band of free 8‐hydroxyquinoline (8‐HQ) (Figure S9, SI). This broad band overlaps with two sharp transition bands, which do not shift with temperature (λ_max_=300 and 314 nm). These transitions could be due to a plethora of phenomena, including LMCT/MLCT transitions, Rayleigh and Raman scattering. A detailed explanation of this phenomenon was, however, beyond the scope of this study. A sharp absorption band (ϵ>1.2) for λ_max_=229 nm was also present (π→π* or n→π*), which however was not taken into account for following the hydrolysis of the ester bond, due to partial overlapping with buffer absorptions (Figure S10, SI). Over 23–26 hours, at least two processes are simultaneously at play in the PBS/DMSO solution of **4**, both at 23 and 37 °C. One is the hydrolysis of the ester bond, the other the shift in the equilibrium between prototropic species of 8‐HQ, as reported for 8‐HQ in aqueous solutions at neutral pH.^39^, ^40^ 8‐HQ alone in PBS/DMSO in fact also showed an evolution profile over time, at 23 and 37 °C, alike (Figure 3, right). For **4**, the bands at λ_max_ 240 and 256 nm, characteristic of the quinoline heterocycle, show a clear evolution profile, either linear or not, depending on the temperature, even after complete hydrolysis. In addition, in the spectrum of **4**, scattering from the ruthenacarborane fragment is evident in the region 240–320 nm (Figure 3, left). At 23 °C hydrolysis of the ester bond proceeds linearly with time (Figure 3, top left), and can be easily monitored following the increase in intensity of the band at λ_max_ 240 nm, and the red shift of the broad band at 250–320 nm to 290–340 nm, which belong to free 8‐HQ (Figure S9, SI). An analogous red shift of the broad extinction band, following hydrolysis, is observed at 37 °C (Figure 3, bottom left). At 37 °C, however, the evolution of the band at λ_max_ 240 nm is not linear with time. At 23 °C hydrolysis is pronounced after 5 h, and is complete after 23 h, whereas at 37 °C, already after 2.5 h hydrolysis has reached completion.

These results thus suggest that the ester bond of **4** undergoes hydrolytic cleavage, in aqueous solution at pH 7.4, which supports the use of an ester bond between the ruthenacarborane fragment and the quinoline group as cleavable linker under physiologic conditions. Based only on these data, hydrolysis rate constants for **4** were not calculated, due to the contribution of two distinct phenomena to the extinction bands at λ_max_ 240 and 256 nm and the scattering component from the ruthenacarborane fragment itself (240–320 nm).

**Figure 4:**
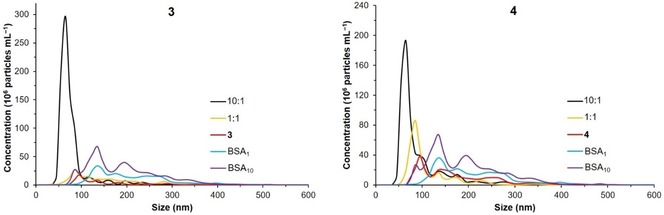
Size distribution of **3** and BSA−**3** (left), and **4** and BSA−**4** (right) in PBS, from NTA measurements. Ratio BSA:metallacarborane was 10 : 1 or 1 : 1. [**3**]=[**4**]=20 μM. [BSA]=20 (BSA_1_) or 200 (BSA_10_) μM. Vol% DMSO is 1 % in all samples. Dilution factors are the same for all samples. Samples were measured 3.5–6 h after preparation. The respective blanks (BSA alone) are also shown. Standard deviation (SD) for particle concentration is ±2.3–3.1×10^7^ (samples with **3**) and ±1.5–2.4×10^7^ (samples with **4**) particles mL^−1^, for particle size ±5–16 nm.

NTA measurements of **3** and **4** in PBS showed that the two complexes spontaneously form self‐assemblies of nanometer size, with high polydispersity and in high concentration (10^6^–10^7^ particles mL^−1^, Figure 4). Upon addition of 10 equivalents of bovine serum albumin (BSA), a significant increase of particle concentration was observed, with respect to **3** (or **4**) and BSA alone (Figure 4). Furthermore, the polydispersity of the samples was greatly reduced to a mostly monomodal dispersion, in an analogous way as we recently extensively described for unsubstituted complexes of the type [3‐(*η* ^6^‐arene)‐3,1,2‐RuC_2_B_9_H_11_].^9^ When changing the molar ratio from 10 : 1 to 1 : 1 (BSA:metallacarborane), the control over size distribution of the self‐assemblies is completely lost in the case of the BSA−**3** system, whereas for BSA−**4** it is partially retained, although the concentration of co‐assemblies is 10× lower than in the corresponding samples with a 9‐fold excess of BSA. This supports our previous investigations, in that an excess of BSA is needed for size stabilization of the BSA−ruthenacarborane co‐assemblies.^9^ The mean particle size for BSA−ruthenacarborane (10 : 1) systems is very similar for the two complexes, with values of 65–70 nm. Based on these results, and on our recent studies on related ruthenacarboranes, we suggest that the spontaneous self‐assembly in aqueous solutions, with and without BSA, is a property of the metallacarborane fragment itself, regardless of the presence (and type) of cluster‐bound substituents.

Thus, free acid **3** and esters **4** and **5** show sufficient chemical stability in wet DMSO for application in cell cultures. Moreover, **3** and **4** showed spontaneous self‐ and co‐assembly behavior with and without BSA, in PBS, suggesting that the “self‐organizing” nanoparticles/nano‐carriers display properties that might be beneficial for targeting tumor tissues which are characterized by a high degree of vasculature, i. e. prone to high degree of angiogenesis, such as GBM.

### 2.3 In vitro Cell Colorimetric Assays

In our previous studies on the biological activity of ruthenacarborane complexes,^8^ we found evidence of insurgence of cytoprotective autophagy in the MCF‐7 cell line after incubation with the complex [3‐{*η* ^6^‐(4‐Me‐1‐COOEt‐C_6_H_4_)}‐3,1,2‐RuC_2_B_9_H_11_]. This was suppressed, when the cells were exposed to dual treatment with the ruthenacarborane and the lysosomal autophagy inhibitor chloroquine (CQ), thus potentiating the moderate anti‐proliferative activity of the ruthenium complex alone. In the present study, the ruthenacarborane and the quinoline fragments are covalently bound (**4** and **5**). Thus, care must be taken when comparing their biological activities with those of our previous studies, even with the same cell line, because the covalent vs. non‐covalent combination of drugs might imply different mechanisms of action in the cell.^44^ Here, we studied the *in vitro* activity of ruthenacarborane **3**, i. e. free acid, and its 8‐hydroxyquinolyl (**4**) and 4‐{(7‐chloroquinolin‐4‐yl)oxy}butanol‐1‐yl (**5**) esters, against a panel of cancer cell lines (B16 mouse melanoma, A375 human skin melanoma, MCF‐7 human breast adenocarcinoma, LN229 human glioblastoma, U251 human glioma). Human fibroblast cell line MRC‐5, murine microglial cell line BV‐2 as well as peritoneal exudate cells (Mph), were chosen as models for non‐malignant cells. Following the NTA results (see above), cell viability assays were performed using two different drug formulations, namely with and without pre‐incubation with BSA, as we reported recently.^9^ On the one side, we wanted to test the possible modulation of the biological activity, depending on dispersity of the nanoparticles (monomodal or polymodal). On the other side, we wanted to assess the reproducibility of the viability data, under either controlled (with BSA) or uncontrolled (without BSA) conditions, in two sets of independent assays. The DMSO stock solutions of complexes **3**–**5** were either directly diluted with cell culture medium to the desired final concentrations (1 to 100 μM), or first incubated for 0.5–1 h with a 9‐fold excess of BSA in PBS, and then diluted with cell culture medium. Cells were exposed to **3**–**5** for 72 h, after which cell viability was determined via 3‐(4,5‐dimethylthiazol‐2‐yl)‐2,5‐diphenyltetrazolium bromide (MTT) and crystal violet (CV) assays, in parallel (Table 2). Cisplatin was also tested as reference. Discrepancies in the calculated IC_50_ values obtained from the two assays (MTT or CV) were found for all tested complexes, as well as for cisplatin against the B16 cell line (Table 2).

**Table 2:** IC_50_ values for **3**–**5** from MTT and CV cell viability assays. Standard deviations for each IC_50_ value are given.

					IC_50_ [μM]				
					Cells				
Compound	Assay	A375	B16	MCF‐7	U251	LN229	BV‐2	MRC‐5	Mph
**3**	MTT	9.7±1.1	19.4±0.9	7.7±0.8	>100	80.1±4.0	64.7±0.7	44.2±1.6	–^b^
	CV	18.7±1.8	21.1±2.0	22.9±1.4	>100	80.0±7.7	68.2±1.3	58.5±0.3	49.1±1.2
BSA−**3** ^a^	MTT	22.5±0.5	23.8±0.1	14.2±2.3	>100	>100	66.7±0.8	35.5±3.6	–^b^
	CV	35.0±7.1	32.0±1.7	23.3±7.9	>100	>100	71.5±1.2	56.3±0.9	50.0±5.0
**4**	MTT	11.9±1.2	20.4±2.1	15.9±0.2	46.2±1.9	42.4±3.9	8.7±0.1	3.9±0.1	–^b^
	CV	14.9±2.1	24.5±2.1	16.9±1.1	45.1±0.8	33.4±3.5	9.2±0.1	4.9±0.1	94.0±7.7
BSA−**4**	MTT	18.4±0.8	15.8±1.0	15.4±0.4	40.4±1.4	37.4±3.2	9.4±0.1	4.3±0.1	‐
	CV	17.2±1.3	21.1±2.7	16.6±0.7	40.7±2.5	31.1±8.1	9.9±0.1	5.0±0.1	100.0±0.1
**5**	MTT	43.4±0.3	9.5±1.8	15.5±1.5	>100	86.7±3.0	12.3±0.1	>100	‐
	CV	>100	33.7±2.9	34.1±0.5	>100	82.5±7.6	>100	>100	>100
BSA−**5**	MTT	39.1±1.9	6.7±1.8	17.4±0.8	>100	84.5±0.7	11.4±0.1	>100	–^b^
	CV	95.1±6.9	32.7±12.9	37.5±3.5	>100	90.2±0.9	>100	>100	>100
Cisplatin	MTT	3.3±0.4	6±0.4	3.0±0.5	0.4±0.1	4.1±0.4	–^b^	–^b^	–^b^
	CV	3.7±0.1	10.9±1.2	3.5±0.2	0.8±0.3	4.1±0.6	–^b^	–^b^	–^b^

^a^ BSA−ruthenacarborane indicates incubation of **3**–**5** with a 9‐fold excess BSA, before dilution with cell culture medium. ^b^ “–“ stands for not tested.

In general, the IC_50_ values for all compounds of the BSA‐free formulations are in good accordance with those for the respective BSA‐stabilized formulations, in both assays (Table 2, Figure S11, SI).

Compound **3** decreased cell viability in a dose‐dependent manner in A375, B16 and MCF‐7 cell lines (Table 2, Figure S11, SI), with values of 18–23 μM (CV). Significant is that, when comparing **3** to the corresponding unsubstituted complex [3‐(*η* ^6^‐*p*‐cymene)‐3,1,2‐RuC_2_B_9_H_11_],^8^ the simple introduction of a carboxylic acid group at a *C*‐vertex of the dicarbollide cluster (**3**) was sufficient to promote sensitization of the B16 cell line to the treatment. At this stage, it is premature to venture an hypothesis on the reason for this, since further studies would be necessary, and also because cellular uptake mechanisms and intracellular binding of boron‐containing drugs in B16 cells still remain poorly understood and are partly controversial.^5^, ^41^ The glioblastoma (LN229) and glioma (U251) cell lines were found to be resistant to treatment with **3**, the free acid (IC_50_>100 μM for U251, 80.0 μM for LN229), and **5**, the alkyl quinoline‐based ruthenacarborane ester (IC_50_>100 μM for U251, 82.5 μM for LN229), for the concentration range tested (Table 2). Conversely, **4**, the aryl 8‐hydroxyquinolyl ester, effectively impaired cell viability of both U251 and LN229 cell lines (Figure S11, SI), at IC_50_ concentrations of 33–45 μM, depending on assay and formulation type (Table 2). For comparison of **4** with a known active compound, U251 and LN229 cells were treated with CQ, already approved in the clinics as autophagy inhibitor for combinatorial treatments for GBM.^28^ As expected, CQ also affected cell viability in both U251 and LN229 cells (Figure S12, SI; IC_50_=30–50 μM), confirming that in the tested glioblastoma and glioma cell lines autophagy inhibition plays a central role in the decrease of cell viability (IC_50_ **3**∼**5**>**4**∼CQ). Furthermore, the type of quinoline ester, either aryl (**4**) or alkyl (**5**), showed major influence on the activity of the drug against U251 and LN229 cells, which could be related to different efficacy of the complexes in disrupting the autophagosomes,^24^ which are formed in the cytoplasm following an autophagic response, similar as observed by Natarajan et al. for a small library of chloroquine derivatives.^15^ Exposure of transformed non‐malignant human fibroblast MRC‐5 and murine microglial BV‐2 cell lines showed similar sensitivity to the treatment with all compounds, which could be ascribed to their high proliferative rate and might indicate that drugs affected cellular proliferation (Table 2, Figure S13, SI).

Treatment of primary non‐malignant mouse macrophages (Mph), that are non‐dividing cells, with **3**–**5** showed higher impairment of cell viability (2× higher) for **3** in comparison to **4** and **5** (Table 2, Figure S13, SI), with IC_50_ values (CV) of 49, 94 and >100 μM for **3**, **4** and **5**, respectively. This suggests a central role of the quinoline fragment of **4** and **5** in promoting cell survival. That a treatment targeting autophagy shows selectivity toward tumor cells rather than healthy cells, is highly desirable, since questions have been raised on the possible side effects of such a therapeutic treatment, because autophagy is a physiological process, which is found in many different (healthy) tissues of the body.^42^ Further studies *in vivo* will be necessary to assess the selectivity of the ruthenacarborane complexes, against a broader panel of healthy cells and tissues.

Overall, viability data showed good reproducibility (standard deviation <10 %) for the three compounds tested, with both drug formulations, i. e. BSA‐free and BSA‐stabilized. The fact that these self‐assembled BSA−ruthenacarborane nano‐carriers do not greatly affect the *in vitro* biological activity of the ruthenacarborane complex itself, is a rare property, when compared to, for example, the engineered PEG‐based nano‐sized delivery systems used by Sadler and co‐workers for loading the poorly water‐soluble half‐sandwich Ru^2+^ and Os^2+^ complexes [(*η* ^6^‐*p*‐cymene)Ru(1,2‐dicarba‐*closo*‐dodecaborane‐1,2‐dithiolate)],^43^ or to Rutherrin, a Ru^2+^ polypyridyl complex (TLD1433) conjugated with transferrin, in a 1 : 1 ratio, which is currently under clinical evaluation as photosensitizer for the treatment of GBM.^44^

It remains to be elucidated, whether the BSA−ruthenacarborane formulation might be beneficial *in vivo*, where formulations of nanoparticles with low polydispersity might enhance cellular uptake, or exploit the EPR effect to promote tumor penetration and selectivity.

### 2.4 Flow Cytometry, Fluorescence Microscopy, Wound Healing Assay

To evaluate the possible mechanism of action of the ruthenacarboranes, flow cytometric analysis was carried out on two physiologically and morphologically different cell lines, namely the MCF‐7 and the LN229 cell lines, with **3** and **4**. Complex **5**, although it showed the lowest toxicity of the series against the mouse macrophages, was not investigated further because it was inefficient against glioma and glioblastoma cell lines.

LN229 was used as model for aggressive, autophagy‐prone malignant GBM,^45^ whereas MCF‐7 was selected for consistency with our previous studies.^8^, ^9^ We aimed at supporting the observations made from *in vitro* colorimetric assays, concerning the effects of the conjugation with a quinoline residue of the ruthenacarborane fragment on proliferation, survival and death mechanisms of the selected cell lines. All investigations discussed below for **3** and **4** were performed using the BSA‐free formulation, unless otherwise stated.

#### 2.4.1 MCF‐7 Cell Line

**Figure 5:**
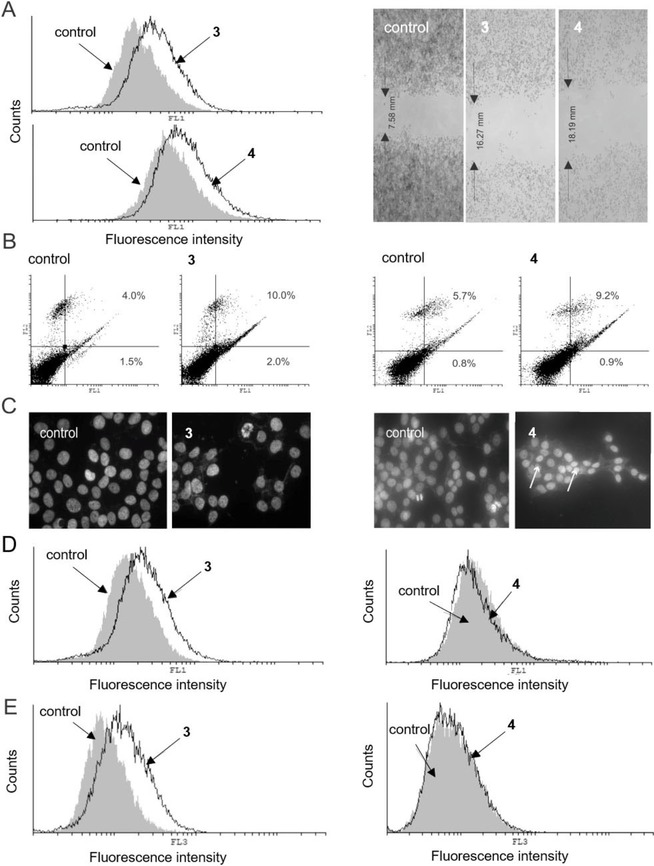
Results from flow cytometric and fluorescence microscopy analysis, and wound healing assay of MCF‐7 cells incubated (72 h) with **3** and **4** at 20 μM. (**A**) CFSE staining (left panel) and wound healing assay (right panel); (**B**) AnnV/PI double staining; (**C**) DAPI‐stained cells observed under fluorescence microscope (magnification X200). Arrows indicate apoptotic cells; (**D**) ApoStat staining; (**E**) AO staining. Experiments were run in triplicate. One representative example per each experiment is shown. For each staining protocol, the respective control (untreated cells) is also shown. (FL1, green channel; FL2, orange channel; FL3, dark red channel).

Treatment of MCF‐7 cells with either **3** or **4** (20 μM, 72 h) showed inhibition of cellular proliferation accompanied by inhibited cell migration, proved by wound healing assay (Figure 5A, left and right panels for **3** and **4**, respectively). In parallel, modest increase of late apoptotic (Ann^+^/PI^+^) cells, with respect to the control (Figure 5B) was observed. When **4** was applied as BSA−**4** formulation, no significant changes were found in the anti‐proliferative activity, compared to the BSA‐free formulation (Figure S16, SI), which supports the observations from the colorimetric assays. However, enhanced granularity of the MCF‐7 cells that was found upon treatment with **4** or BSA−**4** (Figure S17, SI), illustrated that cells efficiently internalized nanoparticles of **4** (or BSA−**4**), or that nanoparticles of **4** bind to the cell surface, similarly as we observed previously for the ruthenium complex [3‐{*η* ^6^‐(biphenyl)}‐3,1,2‐RuC_2_B_9_H_11_].^9^

Confirming our previous data,^8^ microscopic evaluation of DAPI (4′,6‐diamidino‐2‐phenylindole) stained cells showed sparse morphological signs of apoptosis for both **3** and **4** (Figure 5C).

While **3** was found to induce caspase activation, caspase‐independent apoptosis was observed for **4** (Figure 5A and D, right panel), indicating that combination of the quinoline residue with the ruthenacarborane fragment influenced the basic mechanism of action of the ruthenium complex (free acid **3**). This might be due to the interference of autophagic responses with caspase regulation, since caspase‐dependent apoptosis and autophagy are known to share regulatory components, e. g. BCL2 gene, and were also shown to act as mutual inhibitors.^46^ Finally, compound **3** strongly amplified autophagic processes (Figure 5E, left panel). However, in contrast to the complex [3‐{*η* ^6^‐(4‐Me‐1‐COOEt‐C_6_H_4_)}‐3,1,2‐RuC_2_B_9_H_11_], which induced cytoprotective autophagy,^8^ treatment of MCF‐7 cells with **3** and either one of the two inhibitors of autophagy CQ or 3‐methyladenine (3‐MA), resulted in restoration of cellular viability, indicating that in this circumstances autophagy has a cytodestructive role and, thus, contributes to the cytotoxicity of the drug (Figure S15, SI). According to this, compound **4** suppressed autophagy and was less efficient against MCF‐7 cells than **3** (Figure 5E, right panel).

#### 2.4.2 LN229 Cell Line

**Figure 6:**
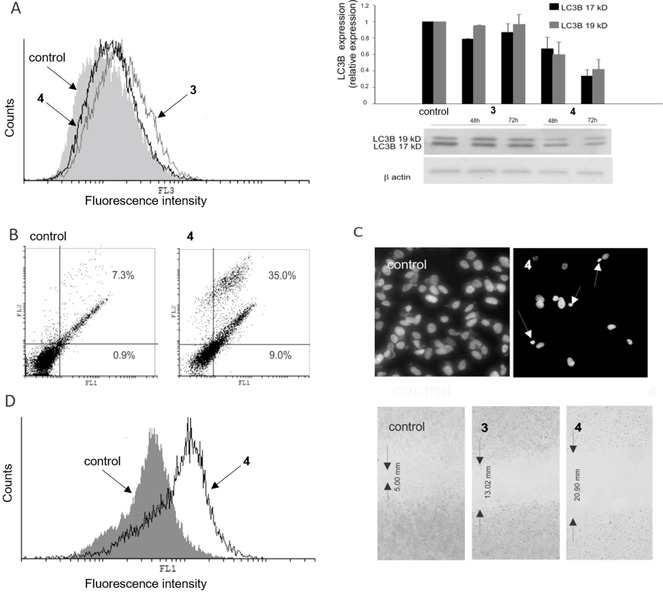
Results from flow cytometric and fluorescence microscopy analysis, wound healing assay and western blot analysis of LN229 cells incubated (72 h) with **4**, at 40 μM. (**A**) AO staining (left panel) and western blot (right panel). Cells incubated (72 h) with **3** are also shown, for comparison; (**B**) AnnV/PI double staining; (**C**) DAPI‐stained cells observed under fluorescence microscope (magnification X200). Arrows indicate apoptotic cells; (**D**) CFSE staining (left panel) and wound healing assay (right panel). Experiments were run in triplicate. One representative example per each experiment is shown. For each staining protocol, the respective control (untreated cells) is also shown. (FL1, green channel; FL2, orange channel; FL3, dark red channel).

When glioblastoma cells (LN229) were treated with an IC_50_ dose of **4** (72 h), the observed number of acidic vesicles was significantly lower than in the same cells treated with an IC_50_ dose of **3** (Figure 6A). Thus, in accordance with the viability data, it is obvious that **4** targets cytoprotective autophagy in LN229 cells. Concordantly, the expression of microtubule‐associated protein light chain 3B (LC3B), which forms a stable association complex with the membrane of autophagosomes, was significantly diminished upon exposure to **4**, confirming once again that **4** efficiently inhibited autophagy.^47^ This result is in accordance with the previous observation by Mauthe et al. that chloroquine inhibits autophagy by impairing autophagosome fusion with lysosomes.^20^ In parallel, an increased number of both early (Ann^+^/PI^−^, from 0.9 % to 9.0 %) and late apoptotic (Ann^+^/PI^+^, from 7.4 % to 35 %) cells were detected after exposure to **4**, with respect to the control (Figure 6B). DAPI staining of LN229 cells incubated with **4** showed several typical morphological features of apoptosis, such as apoptotic bodies, condensed chromatin, shrunken nuclei (Figure 6C). These apoptotic events were accompanied by strong inhibition of cellular proliferation (CFSE staining, Figure 6D, left panel): according to the intensity of fluorescence, cells exposed to **4** stayed undivided, oppositely to control, indicating that inhibition of cellular division precedes cell death. Additionally, cells exposed to **3**, and especially **4**, showed inhibited motility (Figure 6D, right panel). Significantly lower number of cells after the treatment with compounds **3** and **4** was also evident from the scratch test, confirming again the affected proliferative potential besides cell death. Taking this into account, it is not surprising that non‐malignant MRC‐5 and BV‐2 cell lines showed analogous sensitivity to the treatment with **3** and **4** in the viability tests.

Thus, **4** acts *in vitro* as a hybrid molecule against malignant glioblastoma cells (LN229), inhibiting cellular division and, in parallel, cytoprotective autophagy, which supports the rational drug design idea of this work. It is obvious from the recent literature that modulation of autophagy is an attractive therapeutic approach for treatment of GBM, for inducing (re)sensitization to cytotoxic or cytostatic drugs.^12^, ^28^, ^45^, ^48^ It needs to be said that a full elucidation of the cause‐effect relationship between autophagy and cancer cells survival or death would be a very ambitious scope at this point. In fact, the exact mechanisms which trigger and regulate the autophagic response in a specific tissue, healthy or diseased, as well as some steps of the catabolic pathway itself, are today still under dispute, as discussed recently by Yin et al.,^46^ mostly because of the intrinsic difficulty of studying these processes in their “native” state.^49^

## 3 Conclusions

Ruthenacarborane complex **3** was synthesized in two steps from *closo*‐1‐(CH_2_COOH)‐1,2‐C_2_B_10_H_11_ (**1**), in low yield (36 %). The free carboxylic acid group of **3** could be easily activated using Boc_2_O in CH_2_Cl_2_/pyridine to yield ruthenacarborane esters **4** and **5**, after reaction with the respective alcoholate species, the aryl 8‐hydroxyquinolinate (for **4**) and the alkyl 4‐[{(7‐chloroquinolin‐4‐yl)oxy}butan]‐1‐olate (for **5**). The hydrolytic cleavage of the ester bond of **4** at pH 7.4 was complete after 2.5 h (37 °C) or after 23 h (23 °C). The non‐covalent interaction of **3** or **4** with a 9‐fold excess of BSA spontaneously formed nano‐sized particles with low polydispersity, which did not affect the biological activity profiles of **3** and **4** alone. Complex **4** affected cell viability of human glioblastoma and glioma cell lines (LN229 and U251) at concentrations of 33–45 μM, whereas both **3** and **5** were inactive against the same cell lines. This means that both the presence of a quinoline residue and its specific nature (aryl in **4** vs. alkyl in **5**) directly affected the anti‐cancer activity. Moreover, the presence and type of substituents at the *C*‐cluster vertices plays a central role in the biological activity of the ruthenacarborane fragment against the MCF‐7 cell line, being able to affect the basic mechanism of action at the cellular level (see **3** vs. **4** vs. [3‐(*η* ^6^‐*p*‐cymene)‐3,1,2‐RuC_2_B_9_H_11_]). This nicely supports one of the main motivations of using carborane clusters for bioactive compounds, in that substitution at the cluster vertices allows to fine‐tune the properties of potential drug candidates. Flow cytometric analysis on MCF‐7 and LN229 cell lines, together with western blot data, gave convincing evidence that **4** effectively inhibits autophagy, either cytodestructive (in MCF‐7) or cytoprotective (in LN229), and, concomitantly, exerts a strong anti‐proliferative effect on the LN229 cell line, acting thus, in the latter case, as hybrid molecule with dual mode of action *in vitro*.

Thus, **4** is a very promising potential drug candidate for application in the treatment of glioblastomas, since it combines favorable biological activities (anti‐proliferative effects and inhibition of autophagy) with the spontaneous self‐aggregating properties in aqueous solutions, which might be beneficial for selectively targeting angiogenesis‐prone tumors *in vivo*, such as GBM. Compound **4** is now undergoing further evaluation of the biological activity and of the pharmacokinetics of ester hydrolysis in our laboratories, for further assessing its activity in comparison to approved treatments for GBM, and for determining the feasibility of its application *in vivo*.

## Experimental Section

### Methods and Instrumentation

Chemicals were used as purchased. 1‐(CH_2_COOH)‐1,2‐C_2_B_10_H_11_ (**1**) and [(7‐chloroquinolin‐4‐yl)oxy]butanol were synthesized as previously reported,^15^, ^50^ and their purity was assessed via NMR spectroscopy and elemental analysis. Single crystals of **1** were obtained from a saturated diethyl ether solution, at 0 °C, in about three days. Syntheses and characterization data of **6** and **7** are given in the Supplementary Information. All manipulations were carried out in a dry nitrogen or argon atmosphere using standard Schlenk techniques, unless otherwise stated. All manipulations involving thallium(I) compounds were performed wearing personal protective equipment as prescribed in the material safety data sheet (MSDS), and thallium(I)‐containing waste was disposed according to regulations. Solvents were purified and stored as indicated in the Supporting Information. Thin‐layer chromatography (TLC) was carried out on precoated silica glass plates (Merck Silica Gel 60 F254) or on precoated alumina plates (Merck Alumina, pH neutral, 70–230 mesh). Visualization of the compounds on TLC plates was achieved by means of an iodine chamber, or by treatment with a solution of PdCl_2_ (1 wt % in MeOH). Column chromatography was carried out on silica gel (0.035–0.070 mm, 60 Å), or on alumina (pH neutral, 70–230 mesh). Semi‐inert column chromatography was performed using degassed stationary phase and solvents, and flushing the column with nitrogen for 10 min, before column packing.

NMR spectra were acquired at room temperature with a Bruker AVANCE III HD 400 spectrometer. ^1^H (400.13 MHz) and ^13^C{^1^H} (100.16 MHz) NMR spectra were referenced to tetramethylsilane (TMS) as internal standard. ^11^B (128.38 MHz) NMR spectra were referenced to the unified Ξ scale.^51^ For the discussion of NMR data, α and β indicate the two cluster‐bound methylene protons. Mass spectrometry measurements were carried out with an ESI‐MS Bruker ESQUIRE 3000 (Benchtop LC Iontrap) spectrometer, or Synapt G2‐S*i* spectrometer (Waters GmbH, Eschborn, Germany). FT‐IR spectra were obtained with a PerkinElmer system 2000 FTIR spectrometer, scanning between 400 and 4000 cm^−1^. Elemental analyses were performed with a Hereaus VARIO EL oven. X‐ray data for **1** and **3** were collected with a GEMINI CCD diffractometer (Rigaku Inc.), using Mo‐K_α_ radiation (λ=0.71073 Å), T=130(2) K and ω‐scan rotation. Data collection and refinement data are given in Table S1 (SI). Absorption corrections were performed with SCALE3 ABSPACK.^52^ The structures were solved by direct methods (**1**) or by dual‐space methods (**3**), with SHELXS and SHELXT‐2014, respectively.^53^ Structure refinement was done with SHELXL‐2016^54^ by using full‐matrix least‐square routines against *F* ^2^. All non‐hydrogen atoms were refined with anisotropic thermal parameters and the HFIX command was used to locate all hydrogen atoms. The C_2_ unit of the carborane cluster was located with bond length analysis. The pictures were generated with the programs Diamond (version 3.2)^55^ or Mercury (version 3.10).^56^ CCDC 1915974 (**1**) and 1915975 (**3**) contain the supplementary crystallographic data for this paper. UV‐vis absorption spectra were measured with a PerkinElmer UV/VIS/NIR Lambda 900 spectrometer, equipped with tungsten‐halogen and deuterium lamps, using quartz cuvettes (V=3 cm^3^, l=10 mm). Spectra were recorded in the range 240–600 nm, at 1.0 nm resolution. Nanoparticle Tracking Analysis data were recorded using a NanoSight LM10 (Malvern Instruments Ltd, Worcestershire, UK), containing a sample chamber of about 0.25 mL, and equipped with a 532 nm‐laser, a microscope LM14B and a camera sCMOS. The NTA 3.0 analytical software (NanoSight Ltd) was used for both capture and processing. Acquisition and processing parameters were optimized for each sample and the respective blank. Flow cytometry analyses were carried out on a CyFlow® Space instrument (from Sysmex Partec GmbH, Germany), equipped with five lasers (405, 488, 532, 561, 638–640 nm) and a charge‐coupled device (CCD) camera for sample flow monitoring. Results were analyzed with PartecFloMax® software, as reported previously.^57^

### Syntheses

***rac*** **‐[M][*nido*‐7‐(CH_2_COOH)‐7,8‐C_2_B_9_H_11_] (2) (M=K (K[2]) or Na (Na[2]))**

*Method A*. Potassium hydroxide (625 mg, 11.13 mmol, 4.5 eq.) was dissolved in dry EtOH (30 mL). **1** (500 mg, 2.47 mmol, 1.0 eq.) was added in one portion and the mixture was brought to reflux for 21 h. The solvent was then removed *in vacuo*, leaving an off‐white solid. The residue was taken up with H_2_O (4 mL) and acidified to pH 2 with HCl_aq_ (20 vol %). The water phase was concentrated to 1 mL, cooled to 5 °C and filtered. The white solid was washed with *n*‐hexane (10 mL, 15 min sonication), filtered off, redissolved in Et_2_O (13 mL) and filtered through Celite. The volatiles were removed, and the colorless solid was dried *in vacuo* for 12 h (50 °C, 10^−3^ mbar), yielding pure **K[2]** (Yield: 338 mg, 59 %).

*Method B*. Alternatively, **1** (57 mg, 0.28 mmol, 1.0 eq.) was added to EtOH/H_2_O (5 mL, 3 : 2 (v/v)), under nitrogen atmosphere, forming a suspension. NaF (58.8 mg, 1.4 mmol, 5.0 eq.) was added in one portion, and the mixture was heated to 90 °C. Reaction progress was followed via ^11^B{^1^H} NMR spectroscopy. After 18 h, heating was stopped, and the mixture was left to cool down to room temperature. Amberlite (IR120) was added and the mixture was stirred for another hour, then filtered and the solvent evaporated to dryness. The white residue was taken up in Et_2_O (2 mL), filtered through Celite and evaporated to dryness. The crude product was washed three times with *n*‐hexane (3×4 mL, 3×15 min sonication), filtered and dried *in vacuo* for 16 h (50 °C, 10^−3^ mbar), yielding **Na[2]** (Yield: 21 mg, 35 %). NMR data for **Na[2]** are identical to those of **K[2]**.

**K[2]**. ^1^H NMR (CD_3_CN): δ (ppm)=−2.56 (1H, br s, *endo*‐*H*), −0.63–2.48 (br, B−*H*), 1.81 (1H, br s, C_cluster_−*H*), 2.31 (1H, d, ^2^ *J*_HH_=15.9 Hz, *H* ^1α^), 2.59 (1H, d, ^2^ *J*_HH_=16.2 Hz *H* ^1β^). ^11^B NMR (CD_3_CN): δ (ppm)=−10.6 (2B, d, ^1^ *J*_BH_=135 Hz), −14.2 (1B, d, ^1^ *J*_BH_=159 Hz), −17.2 (1B, d, ^1^ *J*_BH_=152 Hz), −18.4 (2B, d, ^1^ *J*_BH_=152 Hz), −22.2 (1B, d, ^1^ *J*_BH_=144 Hz), −33.5 (1B, dd, ^1^ *J*_BH_=127, 49 Hz) −37.2 (1B, d, ^1^ *J*_BH_=135 Hz). ^13^C{^1^H} NMR (CD_3_CN): δ (ppm)=15.6 (s, *C* ^1^), 44.5 (s, *C*_cluster_−H), 66.2 (s, *C*_cluster_), 174.1 (s, *C* ^2^). IR (KBr; selected absorptions): $\tilde{v}$ (cm^−1^)=3436 (br m, ν_OH_), 2963 (m, ν_CHcluster_), 2597 (m, ν_BH_), 2570 (s, ν_BH_), 2529 (s, ν_BH_), 1709 (s, ν_C=O_), 1262 (s, ν_CO_), 1103 (s), 1030 (s), 802 (s, ν_BB_). ESI‐MS (pos.): m/z=483.1948 (100 %, [2 M+Na]^+^). Anal. calcd for C_4_H_13_B_9_O_2_K (231.14): C, 20.93; H, 5.71. Found C, 21.29; H, 5.92.

***closo‐*** **[3‐(*η*^6^‐*p*‐Cymene)‐1‐(CH_2_COOH)‐3,1,2‐RuC_2_B_9_H_10_] (3)**

*Deprotonation of the* nido*‐carborane(‐1) precursor*. **K[2]** (338 mg, 1.47 mmol, 1.0 eq.) was dissolved in dry THF (10 mL) under argon atmosphere, protected from light. The solution was cooled to −35 °C and TlOEt (0.32 mL, 4.40 mmol, 3.0 eq.) was added in one portion, causing immediate precipitation of a bright yellow solid. The mixture was stirred at −35 °C for 30 min, then at room temperature for an additional 1.5 h. Stirring was stopped, the supernatant THF solution was filtered, and the residue was washed with dry EtOH (8 mL) and *n*‐hexane (5 mL). The yellow solid (**Tl[Tl2]**) was dried *in vacuo* for 3 h and used directly, without further purification.

*Complexation reaction*. [{(*η* ^6^‐*p*‐cymene)RuCl(*μ*‐Cl)}_2_] (300 mg, 0.49 mmol, 0.33 eq.) and **Tl[Tl2]** (1.47 mmol, 1.0 eq.) were thoroughly mixed at −65 °C under argon atmosphere, protected from light. Degassed CH_2_Cl_2_ (8 mL) was added and the red‐orange mixture was left stirring for 18 h to warm up to room temperature. HCl_aq_ (20 vol %, 0.1 mL) was added and the mixture was stirred for 40 min, after which Celite was added, and the volatiles were removed *in vacuo*. The residue was purified via column chromatography on silica gel (length=5 cm, diameter=2.5 cm) in air, using *n*‐hexane/ethyl acetate/acetic acid (1 : 1:0.01 (v/v) →1 : 3:0.01 (v/v)→acetone). Two bands were collected, one yellow band containing pure **3** (R_f_=0.22 in *n*‐hexane/ethyl acetate, 1 : 3 (v/v)), one red‐orange band, containing a mixture of *nido*‐carborane **2** and [{(*η* ^6^‐*p*‐cymene)RuCl(*μ*‐Cl)}_2_] (R_f_=0.09 in *n*‐hexane/ethyl acetate, 1 : 3 (v/v)). **3** was obtained as air‐stable pale‐yellow solid (75 mg, 36 %). Recrystallization from chloroform afforded pale yellow platelets, suitable for X‐ray diffraction analysis. **3** is soluble in acetone, THF, acetonitrile and DMSO, moderately soluble in chloroform and dichloromethane.

^1^H NMR (CDCl_3_): δ (ppm)=0.82–3.91 (br, B−*H*), 1.31 (3H, d, ^3^ *J*_HH_=6.9 Hz, *H* ^8^ or *H* ^9^), 1.33 (3H, d, ^3^ *J*_HH_=6.9 Hz, *H* ^8^ or *H* ^9^) 2.35 (3H, s, *H* ^10^), 2.92 (1H, hept, ^3^ *J*_HH_=6.9 Hz, *H* ^7^), 3.23 (1H, d, ^2^ *J*_HH_=16.7 Hz, *H* ^11α^), 3.43 (1H, d, ^2^ *J*_HH_=16.7 Hz, *H* ^11β^), 4.71 (1H, s, C_cluster_−*H*), 5.74 (1H, d, ^3^ *J*_HH_=6.1 Hz, *H* ^2^ or *H* ^6^), 5.87 (1H, d, ^3^ *J*_HH_=6.1 Hz, *H* ^3^ or *H* ^5^), 5.89 (1H, d, ^3^ *J*_HH_=6.2 Hz, *H* ^3^ or *H* ^5^), 5.93 (1H, d, ^3^ *J*_HH_=6.0 Hz, *H* ^2^ or *H* ^6^). ^11^B NMR (CDCl_3_): δ (ppm)=1.6 (2B, d, ^1^ *J*_BH_=138 Hz), −3.4 (1B, d, ^1^ *J*_BH_=147 Hz), −7.8 (1B, d, ^1^ *J*_BH_=144 Hz), −8.9 (1B, d, ^1^ *J*_BH_=137 Hz) −10.0 (1B, d, ^1^ *J*_BH_=143 Hz), −14.4 (1B, d, ^1^ *J*_BH_=169 Hz), −18.3 (1B, d, ^1^ *J*_BH_=163 Hz), −19.7 (1B, d, ^1^ *J*_BH_=182 Hz). ^13^C{^1^H} NMR (CDCl_3_): δ (ppm)=18.8 (s, *C* ^10^), 22.3 (s, *C* ^8^ or *C* ^9^), 23.2 (s, *C* ^8^ or *C* ^9^), 31.3 (s, *C* ^7^), 51.6 (s, *C* ^11^), 54.7 (s, *C*_cluster_−H), 64.6 (s, *C*_cluster_), 88.1 (s, *C* ^3^ or *C* ^5^), 88.5 (s, *C* ^3^ or *C* ^5^), 90.8 (s, *C* ^2^ or *C* ^6^), 91.6 (s, *C* ^2^ or *C* ^6^), 102.1 (s, *C* ^4^), 112.2 (s, *C* ^1^) 175.9 (s, *C* ^12^). IR (KBr; selected vibrations): $\tilde{v}$ (cm^−1^)=3436 (br m, ν_OH_), 2963 (m, ν_CHcluster_), 2597 (m, ν_BH_), 2570 (s, ν_BH_), 2529 (s, ν_BH_), 1709 (s, ν_C=O_), 1262 (s, ν_CO_), 1103 (s), 1030 (s), 802 (s, ν_BB_). ESI‐MS (pos.): m/z=425.2389 (100 %, [M]^+^). Anal. calcd for C_14_H_27_B_9_O_2_Ru (425.73): C, 39.50; H, 6.39. Found C, 39.09; H, 6.20.

***closo*** **‐[3‐(*η*^6^‐*p*‐Cymene)‐1‐(quinolin‐8‐yl‐acetate)‐3,1,2‐RuC_2_B_9_H_10_] (4)**

Compound **3** (63.0 mg, 0.15 mmol, 1.0 eq.) was dissolved in dry CH_2_Cl_2_/pyridine (5.5 mL, 10 : 1 (v/v)) under argon atmosphere and cooled down in an ice bath (H_2_O‐NaCl). Di‐*tert*‐butyl‐dicarbonate (Boc_2_O, 51 μL, 48.4 mg, 0.22 mmol, 1.5 eq.) was added in one portion. After 5 min, the cooling bath was removed, and the mixture was stirred at room temperature for 2 h. In parallel, 8‐hydroxyquinoline (26.9 mg, 0.18 mmol, 1.25 eq.) was dissolved in dry CH_2_Cl_2_ (5.5 mL) and cooled to −35 °C. NaNH_2_ (7.2 mg, 0.18 mmol, 1.25 eq.) was added in one portion. After 5 min the cooling bath was removed, and the mixture was stirred at room temperature for 1.5 h, until gas evolution ceased. The CH_2_Cl_2_/pyridine solution of Boc‐activated acid **3** was then slowly added via cannula to the quinoline‐containing mixture, and the reaction mixture was stirred at room temperature for 19 h. Degassed alumina (ca. 0.5 g) was added to the mixture and volatiles were removed *in vacuo*. The residue was purified via semi‐inert filtration over a short pad of alumina (length=3 cm; diameter=2.5 cm), using degassed CHCl_3_→CHCl_3_/ethyl acetate (7 : 1 (v/v))→ethyl acetate. One yellow band (R_f_=0.93 in CHCl_3_) was collected and evaporated to dryness, yielding **4** as a yellow‐orange powder (15.3 mg, 18 %). **4** was obtained as air‐stable solid, soluble in dichloromethane, chloroform, acetone and DMSO.

^1^H NMR (CDCl_3_): δ (ppm)=0.90–3.80 (br, B−*H*), 1.29 (3H, d, ^3^ *J*_HH_=3.4 Hz, *H* ^8^ or *H* ^9^), 1.31 (3H, d, ^3^ *J*_HH_=3.4 Hz, *H* ^8^ or *H* ^9^), 2.33 (3H, s, *H* ^10^), 2.90 (1H, hept, ^3^ *J*_HH_=6.9 Hz, *H* ^7^), 3.22 (1H, d, ^2^ *J*_HH_=16.6 Hz, *H* ^11α^), 3.42 (1H, d, ^2^ *J*_HH_=16.6 Hz, *H* ^11β^), 4.69 (1H, s, C_cluster_−*H*), 5.72 (1H, d, ^3^ *J*_HH_=6.1 Hz, *H* ^2^ or *H* ^6^), 5.85 (1H, d, ^3^ *J*_HH_=6.1 Hz, *H* ^3^ or *H* ^5^), 5.87 (1H, d, ^3^ *J*_HH_=6.2 Hz, *H* ^3^ or *H* ^5^), 5.90 (1H, d, ^3^ *J*_HH_=6.0 Hz, *H* ^2^ or *H* ^6^), 7.42 (1H, dd, ^3^ *J*_HH_=8.3, 4.2 Hz, *H* ^19^), 7.48–7.54 (2H, m, *H* ^14^ and *H* ^16^), 7.70 (1H, dd, ^3^ *J*_HH_=6.2, 3.4 Hz, *H* ^15^), 8.16 (1H, dd, ^3^ *J*_HH_=8.3, ^4^ *J*_HH_ =1.5 Hz, *H* ^18^), 8.92 (1H, dd, ^3^ *J*_HH_=4.2, ^4^ *J*_HH_=1.5 Hz, *H* ^20^). ^11^B NMR (CDCl_3_): δ (ppm)=1.6 (2B, d, ^1^ *J*_BH_=136 Hz), −3.4 (1B, d, ^1^ *J*_BH_=146 Hz), −7.8 (1B, d, ^1^ *J*_BH_=134 Hz), −8.9 (1B, d, ^1^ *J*_BH_=139 Hz) −9.9 (1B, d, ^1^ *J*_BH_=138 Hz), −14.4 (1B, d, ^1^ *J*_BH_=153 Hz), −18.2 (1B, d, ^1^ *J*_BH_=181 Hz), −19.7 (1B, d, ^1^ *J*_BH_=193 Hz). ^13^C{^1^H} NMR (CDCl_3_): δ (ppm)=18.9 (s, *C* ^10^), 22.3 (s, *C* ^8^ or *C* ^9^), 23.2 (s, *C* ^8^ or *C* ^9^), 31.3 (s, *C* ^7^), 34.9 (s, *C* ^11^), 54.6 (s, *C*_cluster_), 66.5 (s, *C*_cluster_−H), 88.1 (s, *C* ^3^ or *C* ^5^), 88.5 (s, *C* ^3^ or *C* ^5^), 90.9 (s, *C* ^2^ or *C* ^6^), 91.6 (s, *C* ^2^ or *C* ^6^), 102.2 (s, *C* ^4^), 112.2 (s, *C* ^1^), 121.0 (s, *C* ^14^), 121.8 (s, *C* ^16^), 125.7, 126.2, 135.9, 141.3 (s, *C* ^17^), 147.5 (s, *C* ^21^), 150.4 (s, *C* ^20^), 152.1 (s, *C* ^13^), 168.6 (s, *C* ^12^). IR (KBr; selected vibrations): $\tilde{v}$ (cm^−1^)=3439 (w, ν_OH_), 2964 (m, ν_CHcluster_), 2529 (w, ν_BH_), 1754 (s, ν_C=O_), 1369 (m), 1261 (s, ν_CO_), 1148 (s), 1098 (s), 1025 (s), 801 (s, ν_BB_). ESI‐MS(−): m/z=590.4365 (100 %, [M+Cl]^−^). Anal. calcd for C_23_H_32_B_9_NO_2_Ru (555.23): C, 49.97; H, 5.83; N, 2.53. Found C, 50.03; H, 6.02; N, 2.74.

***closo*** **‐[3‐(*η*^6^‐*p*‐Cymene)‐1‐[(7‐chloroquinolin‐4‐yl)oxy]butyl acetate)‐3,1,2‐RuC_2_B_9_H_10_] (5)**

Compound **5** was synthesized in an analogous way as described for **4**, from **3** (100 mg, 0.23 mmol, 1.0 eq.), 4‐{(7‐chloroquinolin‐4‐yl)oxy}butanol (73.9 mg, 0.29 mmol, 1.25 eq.), Boc_2_O (81 μL, 76.9 mg, 0.35 mmol, 1.5 eq.) and NaNH_2_ (11.5 mg, 0.29 mmol, 1.25 eq.). The product was purified as described for **4**, using CHCl_3_→CHCl_3_/acetone (7 : 1 (v/v)). The crude product from the column (yellow‐orange band, R_f_=0.96 in CHCl_3_) was washed with *n*‐hexane (2×3 mL; 2×10 min sonication) to yield pure **5** (32.7 mg, 21 %) as an air‐stable yellow‐orange powder.

^1^H NMR (CDCl_3_): δ (ppm)=0.29–3.45 (br, B−*H*), 1.30 (3H, d, ^3^ *J*_HH_=3.9 Hz, *H* ^8^ or *H* ^9^), 1.32 (3H, d, ^3^ *J*_HH_=4.0 Hz, *H* ^8^ or *H* ^9^), 1.86–2.00 (2H, m, *H* ^14^ or *H* ^15^), 1.99–2.10 (2H, m, *H* ^14^ or *H* ^15^), 2.34 (3H, s, *H* ^10^), 2.91 (1H, hept, ^3^ *J*_HH_=6.9 Hz, *H* ^7^), 3.22 (1H, d, ^2^ *J*_HH_=16.6 Hz, *H* ^11α^), 3.42 (1H, d, ^2^ *J*_HH_=16.6 Hz, *H* ^11β^), 4.18 (2H, t, ^3^ *J*_HH_=6.4 Hz, *H* ^13^), 4.22 (2H, t, ^3^ *J*_HH_=6.1 Hz, *H* ^16^), 4.69 (1H, s, C_cluster_−*H*), 5.73 (1H, d, ^3^ *J*_HH_=6.1 Hz, *H* ^2^ or *H* ^6^), 5.86 (1H, d, ^3^ *J*_HH_=6.1 Hz, *H* ^3^ or *H* ^5^), 5.89 (1H, d, ^3^ *J*_HH_=6.2 Hz, *H* ^3^ or *H* ^5^), 5.92 (1H, d, ^3^ *J*_HH_=6.2 Hz, *H* ^2^ or *H* ^6^), 6.71 (1H, d, ^3^ *J*_HH_=5.3 Hz, *H* ^18^), 7.43 (1H, dd, ^3^ *J*_HH_=8.8, ^4^ *J*_HH_ =1.9 Hz, *H* ^23^), 8.00 (1H, d, ^4^ *J*_HH_=1.8 Hz, *H* ^21^), 8.13 (1H, d, ^3^ *J*_HH_=8.9, *H* ^24^), 8.72 (1H, d, ^3^ *J*_HH_=5.2, *H* ^19^). ^11^B NMR (CDCl_3_): δ (ppm)=1.5 (2B, d, ^1^ *J*_BH_=113 Hz), −3.3 (1B, d, ^1^ *J*_BH_=139 Hz), −7.8 (1B, d, ^1^ *J*_BH_=144 Hz), −8.9 (1B, d, ^1^ *J*_BH_=137 Hz), −10.0 (1B, d, ^1^ *J*_BH_=142 Hz), −14.4 (1B, d, ^1^ *J*_BH_=152 Hz), −18.2 (1B, d, ^1^ *J*_BH_=156 Hz), −19.7 (1B, d, ^1^ *J*_BH_=210 Hz). ^13^C{^1^H} NMR (CDCl_3_): δ (ppm)=18.7 (s, *C* ^10^), 22.2 (s, *C* ^8^ or *C* ^9^), 23.1 (s, *C* ^8^ or *C* ^9^), 27.6 (s, *C* ^14^ or *C* ^15^), 28.0 (s, *C* ^14^ or *C* ^15^), 31.3 (s, *C* ^7^), 54.5 (s, *C*_cluster_), 55.8 (s, *C* ^11^), 66.3 (s, *C* ^13^), 66.4 (s, *C*_cluster_−H), 67.9 (s, *C* ^16^), 88.1 (s, *C* ^2^ or *C* ^6^), 88.4 (s, *C* ^3^ or *C* ^5^), 90.8 (s, *C* ^3^ or *C* ^5^), 91.5 (s, *C* ^2^ or *C* ^6^), 100.9 (s, *C* ^18^), 102.1 (s, *C* ^4^), 112.1 (s, *C* ^1^), 119.8 (s, *C* ^25^), 123.3 (s, *C* ^24^), 126.4 (s, *C* ^23^), 127.9 (s, *C* ^21^), 135.6 (s, *C* ^22^), 149.7 (s, *C* ^20^), 152.5 (s, *C* ^19^), 161.4 (s, *C* ^17^), 168.5 (s, *C* ^12^). IR (KBr; selected vibrations): $\tilde{v}$ (cm^−1^)=3408 (w, ν_OH_), 2924 (m, ν_CHcluster_), 2497 (s, ν_BH_), 1743 (s, ν_C=O_), 1342 (m), 1325 (m), 1274 (s, ν_CO_), 1238 (s, ν_CO_), 1123 (s), 1086 (s), 789 (s, ν_BB_). ESI‐MS(−): m/z=325.1775 (100 %, [4‐(7‐Cl–C_9_H_5_N)‐O‐(CH_2_)_4_‐COOCH_2_+Cl]^−^), 517.2159 (42.5 %, [M–(*p*‐cymene)]^−^). Anal. calcd for C_27_H_39_B_9_ClNO_3_Ru (659.42): C, 49.18; H, 5.96; N, 2.12. Found C, 49.23; H, 5.98; N, 2.20.

### Stability studies

*NMR spectroscopy*. To determine the stability in biocompatible organic solvents, **3**–**5** (each ca. 3 mg) were dissolved in water‐containing DMSO‐d_6_ (0.6 mL) in air at room temperature, and periodically frozen (4–5 °C) and unfrozen. The solutions were analyzed with ^1^H and ^11^B{^1^H} NMR spectroscopy over a period of one month.

*UV‐vis spectroscopy*. Stock solutions of **3**, **4** and 8‐hydroxyquinoline (8‐HQ) in distilled DMSO were freshly prepared before use ([**3**]=2.11 mM; [**4**]=6.56 mM; [8‐HQ]=25.76 mM). An aliquot of the DMSO stock solution of **3**, **4** or 8‐HQ was added to 3 mL PBS solution, so that the final concentration was 20 μM. DMSO content was adjusted to 1 vol % (considering the amount of DMSO from the stock solutions) before addition of the ruthenacarborane, in all samples. Time‐resolved UV‐vis spectra of **4** were measured, over a period of 26 h at room temperature (23±1 °C) and at 37±0.3 °C to follow hydrolysis of the ester bond of **4** at physiologic pH (7.4). PBS/DMSO solutions of **3** and 8‐HQ were also measured, as references. All measurements were corrected by subtracting the blank (PBS+1 vol % DMSO). Experiments were run in duplicate.

*Nanoparticle Tracking Analysis (NTA)*. **3** and **4** were analyzed via NTA to study the modulation of self‐assembly behavior in PBS/DMSO mixture (pH 7.4), with and without BSA, in an analogous way as described previously.^9^ Samples of **3** and **4** in PBS/DMSO were prepared as described above for UV‐vis measurements. The solutions were measured 30 min after preparation. Samples of BSA−**3** and BSA−**4** were prepared with two different BSA:ruthenacarborane ratios, namely 10 : 1 and 1 : 1. Stock solutions of BSA in PBS ([BSA]_PBS_=1.128 mM) were diluted to final concentrations of 200 or 20 μM with PBS (V_fin_=5 mL), then an aliquot of the stock solution of **3** or **4** was added, so that the final concentration of metallacarborane was 20 μM. Content of DMSO was adjusted to 1 vol % (considering the amount of DMSO from the stock solutions) before addition of the ruthenacarborane, in all samples. The solutions of BSA−ruthenacarborane were measured 3.5–6 h after preparation. BSA alone in PBS/DMSO was also measured, as blank, using the same capture and processing parameters as for the respective BSA−metallacarborane samples, for direct comparison. All NTA measurements were performed at 25±0.1 °C. Each sample was measured in five independent captures. The time of each capture was set to 60 s.

### Biological Studies

Cells were cultivated as described in the Supporting Information.

*Preparation of drug solutions*. DMSO stock solutions of **3**–**5**, CQ and 3‐MA were prepared at concentrations of 30 mM (**3**, CQ, 3‐MA) or 50 mM (**4**, **5**) and stored at −20 °C. Stock solution of cisplatin was prepared in DMF (10 mM) immediately before use. A stock solution of BSA in PBS was prepared at 1.28 mM concentration, and stored at 4 °C in the dark. For application to cell cultures, serial dilutions of **3**–**5** or CQ were prepared in the cell culture medium, so that the final concentration of the ruthenacarboranes (or CQ) was 1.6, 3.1, 6.3, 12.5, 25, 50 or 100 μM. Alternatively, **3** or **4** (DMSO stock solution) were added to the BSA solution in PBS, always with a 10 : 1 molar ratio of BSA to ruthenacarborane. The BSA−ruthenacarborane solutions were incubated 0.5–1 h at room temperature, then diluted with cell culture medium to the desired final concentrations. Final concentrations of **3** and **4** were the same as for the BSA‐free formulation. Final DMSO was 0.02–0.5 vol %.

*Colorimetric assays for cellular viability*. Cells were exposed to various concentrations (0–100 μM) of **3**–**5** (or BSA−**3**/**4**), CQ or cisplatin for 72 h. For counting the number of attached (viable) cells, cells were fixed with 4 % (w/v) paraformaldehyde for 10 min at room temperature, and subsequently stained for 15 min with 1 mol % crystal violet (CV) solution. Cells were then washed with tap water, dried in air, and the CV dye was dissolved in 33 % (w/v) acetic acid solution. For the detection of mitochondrial respiration, cells were cultivated in MTT staining solution (0.5 mg mL^−1^) for approximately 1 h. The dye was then discarded, and the formed formazan (purple) was dissolved in DMSO. The absorbance was measured with an automated microplate reader at λ_max_ 540 nm, with the reference λ_max_ 670 nm. Untreated cells or cells treated only with BSA (control) were also measured, as reference for BSA‐free and BSA‐containing drug formulations, respectively. For determination of the role of autophagy specific inhibitors, CQ or 3‐MA, were applied at a concentration of 20 μM and 0.5 mM, respectively, and the cells were stained with CV dye. Cell viability is expressed as percentage (%) relative to control (untreated cultures).^58^ Experiments were run in three independent replicates. Standard deviations of the calculated IC_50_ mean values were within 10 %. Non‐linear regression analyses of the obtained results was done using GraphPad Prism software to calculate IC_50_ values.

*Flow cytometry*. For gaining better insights into the mechanisms of action of the ruthenium complexes, MCF‐7 and LN229 cells incubated with an IC_50_ dose of **3** or **4**, were analyzed via flow cytometry. Several staining protocols were carried out in parallel, in independent experiments: i) AnnV/PI for the detection of apoptotic cell death, ii) AO for the detection of acidic vacuoles, iii) ApoStat for checking caspase activation, iv) CFSE for detecting interference with cellular proliferation, and v) DAPI for the detection of morphological signs of apoptosis. Untreated cells or cells treated only with BSA (control) were also measured, as reference for BSA‐free and BSA‐containing drug formulations, respectively. **4** was applied to the MCF‐7 cells as either BSA‐containing or BSA‐free formulation. Staining protocols are described in the Supporting Information. Channels FL1 (green emission), FL2 (orange emission) and/or FL3 (dark red emission) were used for fluorescence detection, according to the specific staining agent. Sideward Scatter (SSC) for MCF‐7 cells treated with **4** or BSA−**4** was also analyzed, with respect to the control, for detection of shifts of cell granularity. Experiments were run in three independent replicates.

*Statistical analysis*. Analysis of variance (ANOVA) followed with a Student‐Newman–Keuls test was used for significance of the differences between treatments, and a *p* value less than 0.05 was taken as statistically significant.

## Conflict of interest

The authors declare no conflict of interest.

## Supporting information

As a service to our authors and readers, this journal provides supporting information supplied by the authors. Such materials are peer reviewed and may be re‐organized for online delivery, but are not copy‐edited or typeset. Technical support issues arising from supporting information (other than missing files) should be addressed to the authors.

Supplementary
