# Supplementary material for: Quinoline‐Conjugated Ruthenacarboranes: Toward Hybrid Drugs with a Dual Mode of Action
Source: ChemMedChem. 2019 Nov 19;14(24):2061–74. doi: 10.1002/cmdc.201900349 (PMC6973020; doi:10.1002/cmdc.201900349)
Supplement: Supplementary file 1 — Supplementary [file CMDC-14-2061-s001.pdf]

CHEMMEDCHEM

Supporting Information

© Copyright Wiley-VCH Verlag GmbH & Co. KGaA, 69451 Weinheim, 2019

Quinoline-Conjugated Ruthenacarboranes: Toward Hybrid Drugs with a Dual Mode of Action

Marta Gozzi, Blagoje Murganic, Dijana Drača, John Popp, Peter Coburger, Danijela Maksimović-Ivanić, Sanja Mijatović, and Evamarie Hey-Hawkins*©2019 The Authors. Published by Wiley-VCH Verlag GmbH & Co. KGaA.
This is an open access article under the terms of the Creative Commons Attribution License, which permits use, distribution and reproduction in any medium, provided the original work is properly cited.

Supporting Information

Table of Contents

1. Notes on Materials Storage and Purification 2
2. Syntheses 2
3. Biological Studies 4
4. NMR Spectra 6
5. Crystallographic Data 10
   4.1. Data Collection and Refinement 10
   4.2. Hydrogen Bonding in **1** and **3** 11
6. UV-vis Spectra 12
7. Cell Viability 14
8. Flow Cytometry 17

9. Fluorescent microscopy

10. Wound healing assay

11. Western blot analysis

9. List of Abbreviations 18

1. Notes on Materials Storage and Purification

Dulbecco`s phosphate-buffered saline (PBS, 10 mM) was purchased from Biowest (w/o Mg and w/o Ca ions; Product Code: L0615), or freshly prepared in our laboratories according to the technical specification sheet from Dulbecco®, and stored at room temperature. PBS used for UV-vis experiments was degassed and stored under nitrogen. Magnesium- and fatty acids-free bovine serum albumin (BSA) was purchased from SERVA electrophoresis GmbH, and stored at –20 °C.
Thallium(I) ethanolate (Alfa Aesar©) was stored under argon at –20 °C, protected from light. Due to the high instability towards hydrolysis, this compound was used no longer than a month from purchase.
Dried and degassed dichloromethane (CH_2Cl_2) and *n*-hexane were obtained from an MBRAUN solvent purification system (MB SPS-800) and stored under nitrogen over molecular sieves (4 Å). Tetrahydrofuran (THF) was dried over Na/benzophenone, freshly distilled prior to use and stored under nitrogen over molecular sieves (4 Å). Pyridine and dimethylsulfoxide (DMSO) were degassed with nitrogen, dried over CaH_2, freshly distilled prior to use and stored under nitrogen over molecular sieves (4 Å). Ethanol was degassed with nitrogen, dried over magnesium, freshly distilled prior to use and stored under nitrogen atmosphere over molecular sieve (4 Å), for the synthesis of **2**. Alternatively, ethanol was distilled, and the EtOH/H_2O 3:2 (v/v) mixture for the synthesis of **7**, was degassed and stored under argon. BSA stock solutions in PBS were stored at 4 °C in the dark.

2. Syntheses

***closo*-1-(Quinolin-8-yl-acetate)-1,2-$C_2B_{10}H_{101}$ (6)**

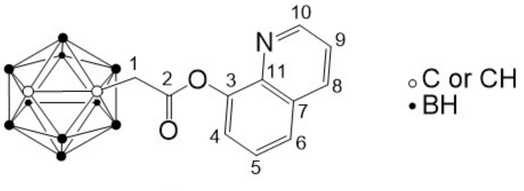

6

6 was synthesised in an analogous way as described for **4** and **5**, from **1** (500 mg, 2.47 mmol, 1.0 eq.), 8-hydroxyquinoline (449 mg, 3.09 mmol, 1.25 eq.), Boc_2O (0.85 mL, 809 mg, 3.71 mmol, 1.5 eq.) and $NaNH_2$ (121 mg, 3.09 mmol, 1.25 eq.). Purification of the crude product was carried out as follows. The reaction mixture was diluted with CH_2Cl_2 (25 mL) and transferred to a separation funnel. The organic phase was washed with water (2 x 25 mL) and sat. aq. $NaHCO_3$ (2 x 25 mL). The water phase was extracted with CH_2Cl_2 (2 x 25 mL), and the combined organic phases were dried over $MgSO_4$. Celite was added and the volatiles were removed with a rotary evaporator. The product was purified via silica-gel column chromatography (length = 13 cm; diameter = 3.5 cm), using *n*-hexane/ethyl acetate 1:1 → 1:3 (v/v), then acetone. The fraction containing **6** (R_f = 0.18, *n*-hexane/ethyl acetate 1:1 (v/v)) was evaporated to dryness, and the residue was recrystallised twice from hot *n*-hexane (50 °C), yielding **6** as a colourless powder (152 mg, 19%). **6** is air-stable in the solid state, but the ester bond slowly hydrolyses in wet acetone or DMSO solutions in air (colour change from yellow to dark green).

^{1}H NMR ($CDCl_3$): δ (ppm) = 1.36–3.04 (br, B–*H*), 3.13 (2H, s, H^1), 4.45 (1H, br s, $C_{cluster}$–*H*), 7.19 (1H, dd, $^{3}J_{HH}$ = 7.6 Hz, $^{4}J_{HH}$ = 1.2 Hz, H^4), 7.34 (1H, dd, $^{3}J_{HH}$ = 8.3 Hz, $^{4}J_{HH}$ = 1.2 Hz, H^7), 7.42–7.49 (2H, m, H^5 and H^9), 8.17 (1H, dd, $^{3}J_{HH}$ = 8.3 Hz, $^{4}J_{HH}$ = 1.6 Hz, H^2), 8.79 (1H, dd, $^{3}J_{HH}$ = 4.3 Hz, $^{4}J_{HH}$ = 1.6 Hz, H^{10}). ^{11}B{^{1}H} NMR ($CDCl_3$): δ (ppm) = −2.2 (1B, s), −5.1 (1B, s), −9.4 (2B, s), −10.5 (2B, s), −12.0 (2B, s), −12.9 (2B, s). ^{11}B NMR ($CDCl_3$): δ (ppm) = −2.2 (1B, d, $^{1}J_{BH}$ = 149 Hz), −5.1 (1B, d, $^{1}J_{BH}$ = 140 Hz), −9.3 (2B, d, $^{1}J_{BH}$ = 147 Hz), −11.8 (2B, d, $^{1}J_{BH}$ = 141 Hz), −13.1 (2B, d, $^{1}J_{BH}$ = 122 Hz).[1] ^{13}C{^{1}H} NMR ($CDCl_3$): δ (ppm) = 42.5 (s, C^1), 58.5 (s, $C_{cluster}$–H), 66.6 (s, $C_{cluster}$–H), 83.4 (s, C^7), 94.9 (s, C^{11}), 98.6 (s, C^3), 110.0 (s, C^4), 117.9 (s, C^6), 121.8 (s, C^5), 127.8 (s, C^9), 136.2 (s, C^8), 147.8 (s, C^{10}), 166.5 (s, C^2). ESI-MS(+): m/z = 354.8611 (100%, $[M+Na]^+$).

***rac*-Na[*nido*-7-(quinolin-8-yl-acetate)-7,8-$C_2B_9H_{11}$] (7)**

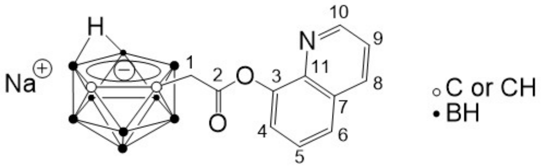

7

7 was synthesised in an analogous way as described for **Na[2]**, from **6** (100 mg, 0,30 mmol, 1.0 eq.) and NaF (66 mg, 1.57 mmol, 5.23 eq.). **7** (79 mg, 64%) was obtained as air-stable, bright yellow powder. **7** is soluble in acetonitrile, acetone and DMSO.

^{1}H NMR ($CDCl_3$): δ (ppm) = −2.93 (1H, br s, *endo-H*),[2] −0.48 to 2.98 (br, B–*H*), 1.92 (1H, br s, $C_{cluster}$–*H*), 2.42 (1H, d, $^{2}J_{HH}$ = 15.9 Hz, $H^{1\alpha}$),[3] 2.65 (1H, d, $^{2}J_{HH}$ = 15.9 Hz, $H^{1\beta}$), 7.25 (1H, d, $^{3}J_{HH}$ = 8.3 Hz, H^4), 7.41 (1H, d, $^{3}J_{HH}$ = 8.2 Hz, H^5), 7.51 (1H, d, $^{3}J_{HH}$ = 8.2 Hz, H^6), 7.55 (1H, dd, $^{3}J_{HH}$ = 8.4, 4.4 Hz, H^9), 8.30 (1H, d, $^{3}J_{HH}$ = 8.4, H^8), 8.85 (1H, d, $^{3}J_{HH}$ = 4.4, H^{10}). ^{11}B NMR ($CDCl_3$): δ (ppm) = −11.3 (1B, d, $^{1}J_{BH}$ = 144 Hz), −12.4 (1B, d, $^{1}J_{BH}$ = 135 Hz), −13.7 (1B, d, $^{1}J_{BH}$ = 186 Hz), −15.0 (1B, d, $^{1}J_{BH}$ = 139 Hz), −18.2 (1B, d, $^{1}J_{BH}$ = 144 Hz), −19.3 (1B, d, $^{1}J_{BH}$ = 130 Hz), −20.3 (1B, d, $^{1}J_{BH}$ = 133 Hz), −32.4 (1B, dd, $^{1}J_{BH}$ = 131, 37 Hz), −36.7 (1B, d, $^{1}J_{BH}$ = 140 Hz). ^{13}C{^{1}H} NMR ($CDCl_3$): δ (ppm) = 28.1 (s, C^1), 45.4 (s, $C_{cluster}$–H), 55.9 (s, $C_{cluster}$), 81.7 (s, C^{11}), 111.3 (s, C^4), 118.3 (s, C^5), 121.9 (s, C^9), 128.3 (s, C^6), 128.8 (s, C^7), 137.9 (s, C^8), 147.2 (s, C^{10}), 151.3 (s, C^3), 175.2 (s, C^2). IR (KBr; selected vibrations): $\tilde{\nu}$ (cm^{-1}) = 3580 (m, ν_{OH}), 2968 (m, $\nu_{CHcluster}$), 2524 (s, ν_{BH}), 1704 (m, $\nu_{C=O}$), 1604 (m, $\nu_{C=O}$), 1371 (m), 1261 (m, ν_{CO}), 1158 (s), 1094 (s), 1026 (s), 820 (s, ν_{BB}), 803 (s, ν_{BB}). ESI-MS(+): m/z = 367.7652 (100%, $[M+Na]^+$). Anal. calcd for $C_{13}H_{20}B_9NNaO_2$ (344.22): C, 45.58; H, 5.88; N, 4.09. Found C, 45.47; H, 5.98; N, 4.29.

---

[1] Not all $^{1}J_{BH}$ constants could be determined, due to extensive overlapping of ^{11}B signals.

[2] *endo*-H is the fluxional extra proton on the C_2B_3 face of the *nido*-carborane(−1) derivatives.

[3] α and β indicate the two cluster-bound methylene protons.

Reaction of 7 with [{(η^6-*p*-cymene)RuCl(μ-Cl)}$_2$]

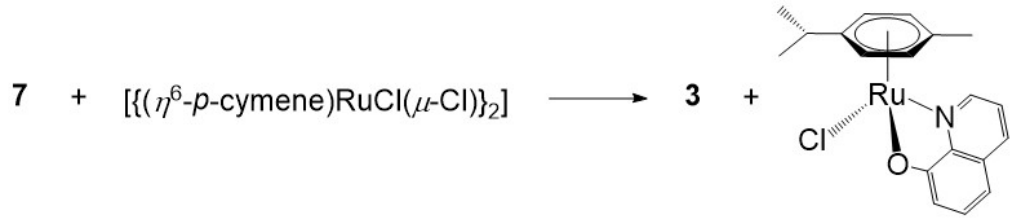

Method A. **7** (115 mg, 0.36 mmol, 1.0 eq.) was dissolved in dry THF (8 mL) and cooled to –30 °C, under argon atmosphere and protected from light. TlOEt (53.9 µL, 189.7 mg, 0.76 mmol, 2.1 eq.) was added in one portion, and the mixture was stirred at –30 °C for 30 min, then for 2 h at room temperature. The solution was concentrated *in vacuo*, to a volume of ca. 2 mL. NMR analysis ($CDCl_3$) of the reaction mixture showed quantitative deprotonation of **7** to its dithallium salt (**Tl[Tl7]**), without cleavage of the ester bond (Figure S2 and Figure S3). The rest of the solvent was evaporated *in vacuo*, yielding a bright yellow residue, which was washed with EtOH (2 x 2 mL), filtered and dried *in vacuo*. [{(η^6-*p*-cymene)RuCl(μ-Cl)}$_2$] (110 mg, 0.18 mmol, 0.5 eq.) was added to the **Tl[Tl7]** salt, the reaction flask was cooled to –65 °C, and degassed CH_2Cl_2 (10 mL) was added. The reaction mixture was left stirring to warm up to room temperature over 16 h. The reaction mixture was then filtered, and the precipitate was identified via NMR spectroscopy as chlorido(8-quinolinato-κ^{2}N,O)(η^6-*p*-cymene)ruthenium(II) (Figure S1). The filtrate was purified via column chromatography on silica gel (dry load with celite), yielding pure complex **3**. The desired ester-substituted ruthenacarborane complex could not be isolated.

Method B. Alternatively, **7** (375 mg, 1.18 mmol, 1.0 eq.) was reacted with lithium bis(trimethylsilyl)amide (LiHMDS, 1M sol. in THF, 1.42 mL, 1.42 mmol, 1.2 eq.), as base, at –78 °C for 1 h, then at room temperature for 40 min. After solvent removal and washing with *n*-hexane (5 mL), the **Na[Li7]** salt was redissolved in 11 mL THF, cooled to –78 °C, and [{(η^6-*p*-cymene)RuCl(μ-Cl)}$_2$] (362 mg, 0.59 mmol, 0.5 eq.) was added in one portion. After 40 min, the cooling bath was removed, and the mixture was left stirring at room temperature for 15 h. The ensuing precipitate was identified once again as chlorido(8-quinolinato-κ^{2}N,O)(η^6-*p*-cymene)ruthenium(II). The filtrate was purified via inert column chromatography on silica gel, yielding only complex **3**.

3. Biological Studies

Reagents and Cells. Fetal calf serum (FCS), phosphate-buffered saline (PBS), dimethylsulfoxide (DMSO), 3-(4,5-dimethylthiazol-2-yl)-2,5-diphenyltetrazolium bromide (MTT), carboxyfluorescein-succinimidyl ester (CFSE), 3-methyladenine (3-MA), chloroquine (CQ) diphosphate salt, crystal violet (CV) and acridine orange (AO) were obtained from Sigma Aldrich (St. Louis, MO, USA). 4',6-diamidino-2-phenylindole (DAPI) was from Fluoromounth-G (Southern Biotech., Birmingham, AL, USA). Cell culture medium RPMI-1640 was from Biowest (Riverside, MO, USA), while Dulbecco's Modified Eagle Medium (DMEM) was from Biological Industries (Cromwell, CT, USA). Annexin V-FITC (AnnV) was from BD Pharmingen (San Diego, CA,USA), while ApoStat was bought from R&D Systems (Minneapolis, MN, USA). Cell lines (human skin melanoma A375, mouse melanoma B16, human breast adenocarcinoma MCF-7, human normal fibroblasts MRC-5, human malignant glioblastoma LN229 and glioma U251) were purchased from the American Type Culture Collection (ATCC, Manassas, Virginia, USA), while

mouse microglial cell line BV-2 was a kind gift from Dr. Irena Lavrnja, Institute for Biological Research “Siniša Stanković” (University of Belgrade). Cells were routinely cultivated in HEPES-buffered RPMI-1640 (A375, B16, MCF-7, MRC-5, BV-2) or DMEM (U251, LN229) medium, supplemented with 10%-FCS, 2 mM L-glutamine, 0.01% sodium pyruvate and antibiotics, at 37 °C in a humidified atmosphere with 5% CO_2. Peritoneal resident macrophages (Mph) were collected from C57BL/6 mice, obtained from the animal facility at the Institute for Biological Research “Siniša Stanković” (University of Belgrade), via peritoneal lavage with ice-cold PBS. Cells were counted and seeded in 96-well plates and incubated overnight, as described above. Before treatment, non-adherent cells were removed. Isolation of cells from animals were performed in accordance to local guidelines and approved by the local Institutional Animal Care and Use Committee (IACUC).

Staining Protocols for Flow Cytometric Analysis. For AnnV/PI, AO and ApoStat staining, cells were treated with an IC_{50} dose of **3** or **4** for 72 h, then detached with trypsin and washed with PBS. In the case of Ann/PI double staining and Apostat, cells were stained according to the manufacturer’s protocols. Cells were incubated with AnnV and PI in Ann-binding buffer (15 µg mL^{-1}; 15 min, room temperature)., or ApoStat (ZVAD-FMK-FITC, 1 µL) in PBS (30 min, 37 °C). For detection of acidic vacuoles, upon treatment cells were stained with AO dye (1 µg mL^{-1}; 15 min, 37 °C), in PBS. Finally, cells were washed in PBS, resuspended in PBS (or AnnV-binding buffer for AnnV/PI), and analysed. For CFSE staining, detached cells were first stained with a PBS solution of CFSE (1 µM; 10 min, 37 °C), then washed, seeded and then exposed to an IC_{50} dose of **3** or **4** for 72 h. Cells were then washed, trypsinised, dissolved in PBS and analysed by CyFlow® Space Partec using the PartecFloMax® software (Partec GmbH, Münster, Germany).

Staining Protocols for Fluorescence Microscopy. To evaluate the presence of apoptosis, cells were seeded in 8-chamber slides at 1.5×10^4 cells/well and treated with an IC_{50} dose of **3** and **4** for 72 h. At the end of the incubation period, cells were fixed with 4% (w/v) paraformaldehyde for 15 min at room temperature, washed and covered with DAPI fluoromounth-G (Southern Biotech, Birmingham, AL, USA), before analysis. Slides were analysed with Zeiss AxioObserver Z1 inverted fluorescence microscope (Carl Zeiss AG, Oberkochen, Germany) at 400× magnification. Morphological signs of apoptosis (irregular nuclei shape, condensed chromatin, apoptotic bodies) were examined by three independent observers.

Wound Healing Assay. For evaluation of the cells’ motility, wound healing assay, in which cells migrate bidirectionally from the edges of a scratch wound, was used. Cells were seeded in 6-well plates, till reaching the confluence, when scratch over cellular layer was drawn with a sterile pipette tip across the centre of the wells to make a clean wound area. Cells were washed twice with fresh PBS to remove loose or damaged cells, and then exposed to an IC_{50} dose of **3** and **4** for 72 h. At the end of the incubation period, cells were washed, fixed with 4% (w/v) paraformaldehyde for 10 min and stained with 2% CV solution for 1 min at room temperature. The wounds were digitally photographed using a Moticam 2000 camera and Nikon Digital Sight Fi2 camera attached to a Nikon SMZ800 stereo zoom microscope.

Western Blot Analysis. Cells were incubated with an IC_{50} dose of **3** or **4** for 48 and 72 h and lysed in protein lysis buffer (62.5 mM Tris–HCl (pH 6.8 at 25 ◦C), 2% (w/v) SDS, 10% glycerol, 50 mM

dithiothreitol). Electrophoretical separation was done on 15% SDS-polyacrylamide gels. As a protein molecular weight marker, PageRuler prestained ladder (Thermo Scientific, USA) was used. Electrotransfer to polyvinylidenedifluoride membranes at 5 mA cm^{-2} was done with a semidry blotting system (Fastblot B43; BioRad, Göttingen, Germany). Membranes were blocked with 5% (w/v) BSA in PBS for 1 h at room temperature. Incubation with specific antibodies for the microtubule-associated protein light chain 3B (LC3B) (Sigma Aldrich, St. Louis, MO, USA) or β-actin (Abcam, Cambridge, UK) was done overnight at 4 °C. As a secondary antibody, goat anti-rabbit IgG-horseradish peroxidase (Santa Cruz Biotechnology, Dallas, TX, USA) was used. Bands were detected using a chemiluminescence detection system (ECL; GE Healthcare, Chalfont St. Giles, Buckinghamshire, UK), while the protein levels were determined by densitometry, using ImageJ software (NIH, Bethesda, MD, USA), and expressed relative to β-actin. The results are presented as fold variation in signal intensity relative to the control, that was arbitrarily set to a value of 1.

4. NMR Spectra

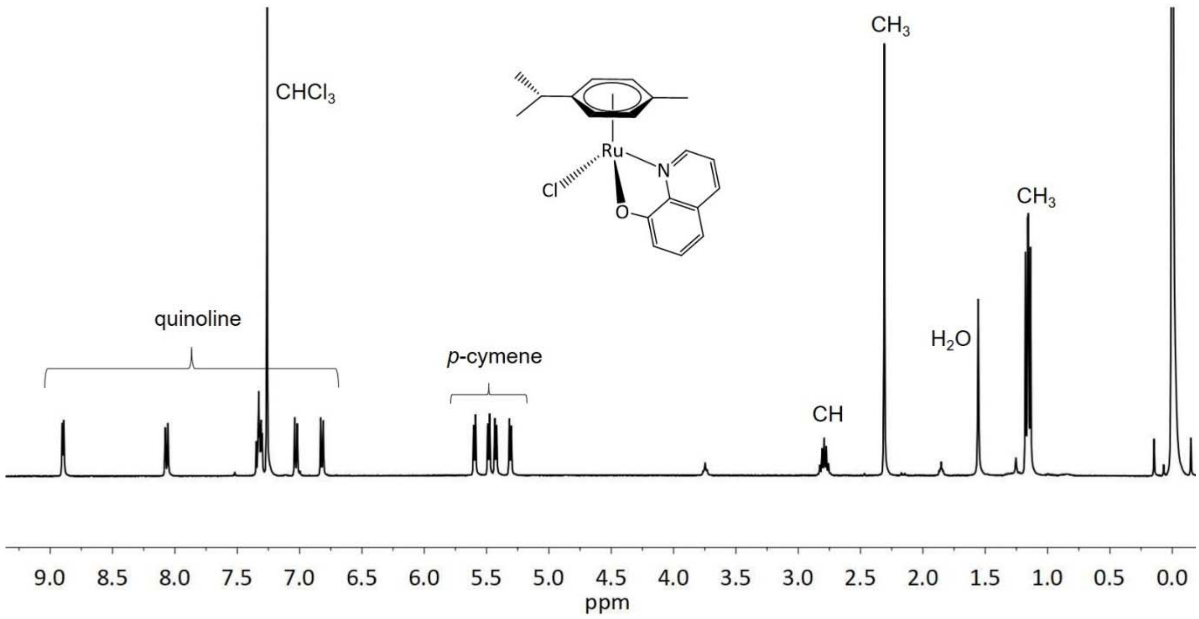

Figure S1. ^{1}H NMR (400.13 MHz) spectrum in $CDCl_3$ of the side product of the reaction of **Tl[Tl7]** with [{(η^6-*p*-cymene)RuCl(μ-Cl)}$_2$], identified as chlorido(8-quinolinato-κ^{2}N,O)(η^6-p-cymene)ruthenium(II).

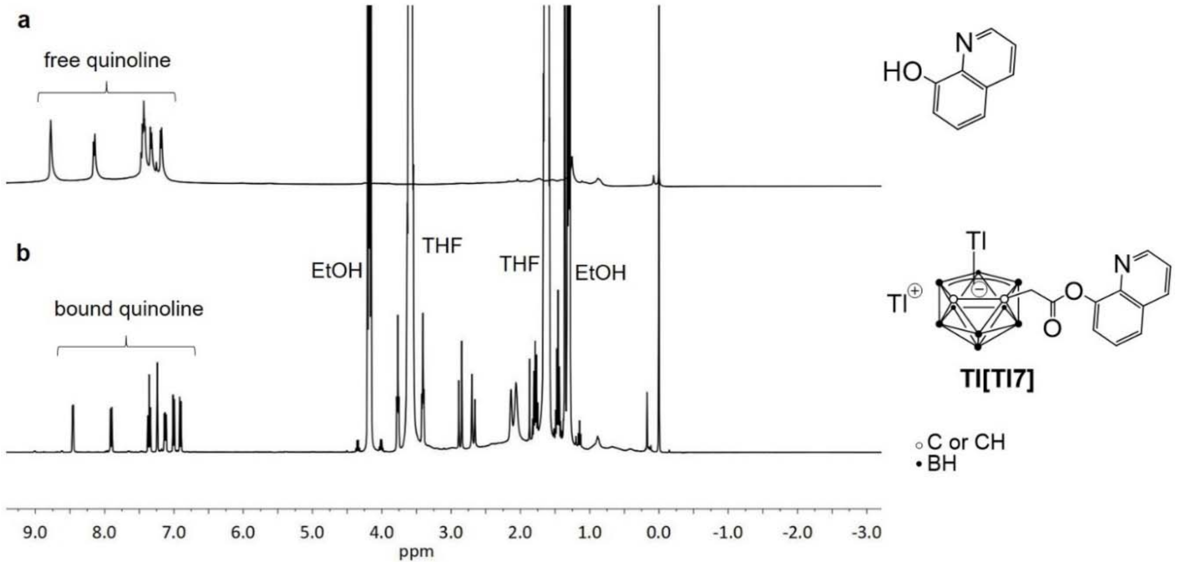

Figure S2. ^{1}H NMR (400.13 MHz) spectra of 8-hydroxyquinoline (**a**) and **Tl[Tl7]** (**b**) in $CDCl_3$. Aromatic signals for free and bound quinoline are shown.

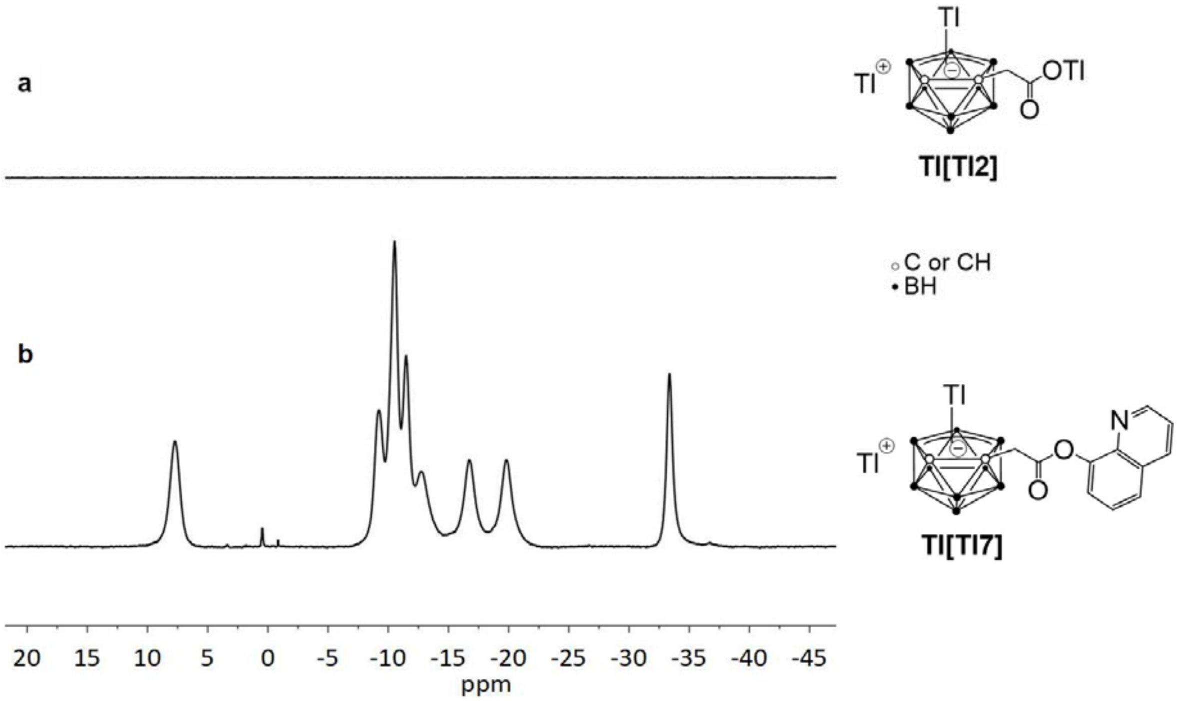

Figure S3. $^{11}B\{^{1}H\}$ NMR spectra (128.38 MHZ) of **Tl[Tl2]** (**a**) and **Tl[Tl7]** (**b**) in $CDCl_3$. No ^{11}B signals for **Tl[Tl2]** could be detected due to low solubility, while **Tl[Tl7]** was soluble enough in $CDCl_3$ to give a proper spectrum.

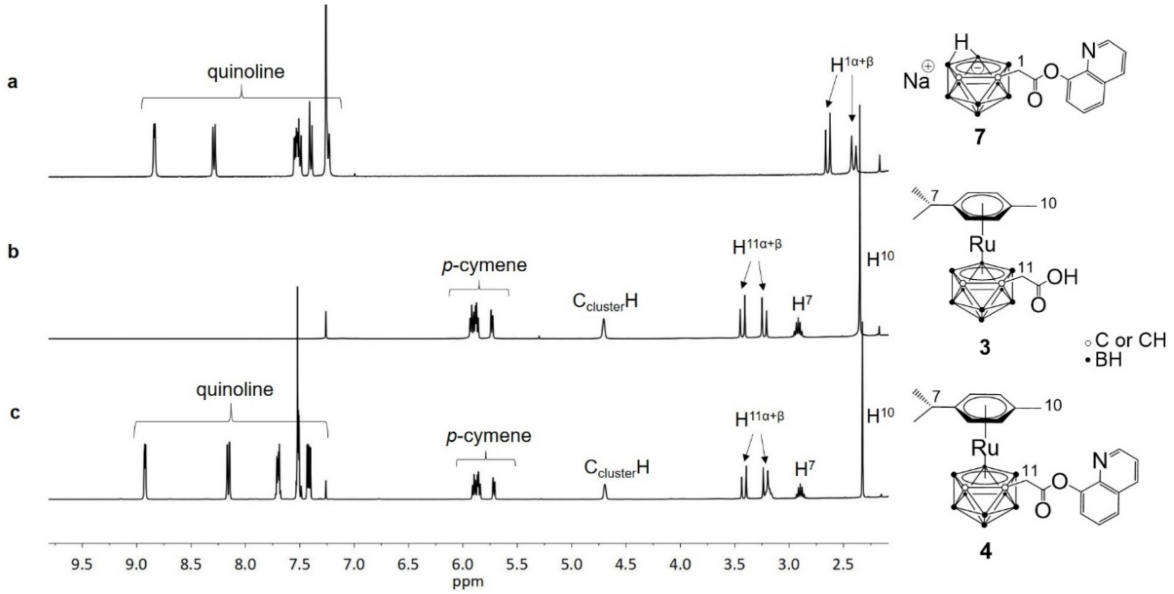

Figure S4. ^{1}H NMR spectra (400.13 MHz) of **7** (**a**), **4** (**b**) and **3** (**c**) in $CDCl_3$. Assignment of selected signals is given. α and β refer to the two cluster-bound methylene protons.

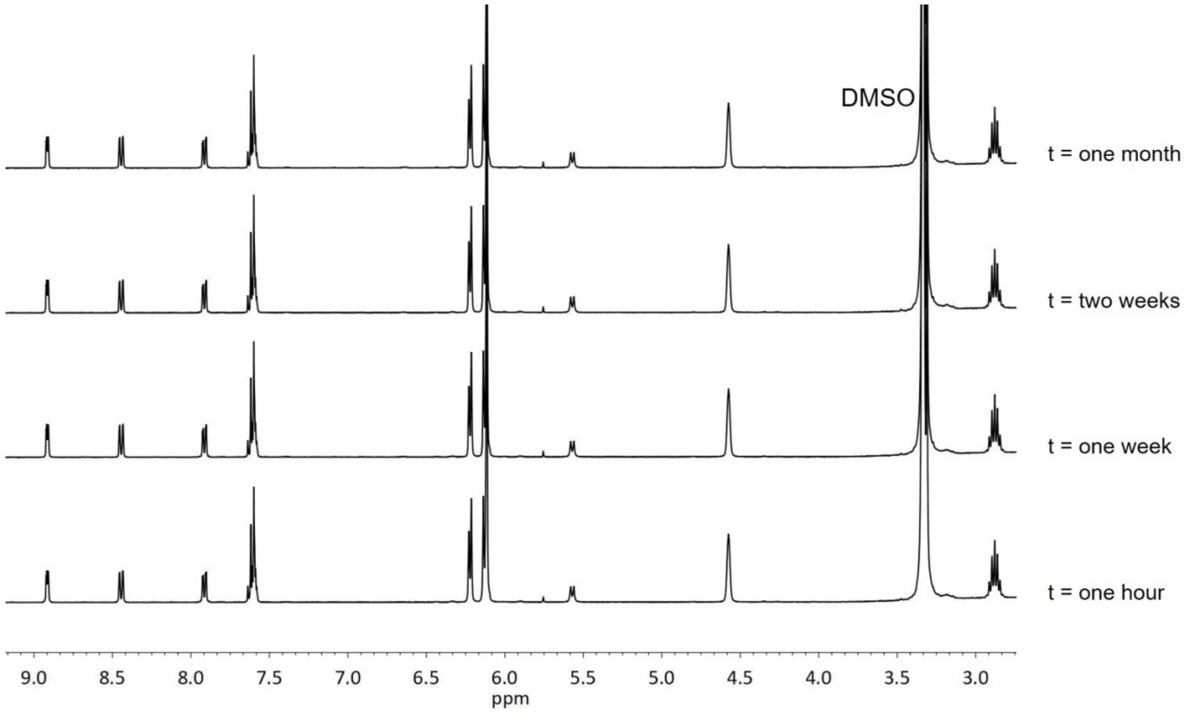

Figure S5. ^{1}H NMR spectra (400.13 MHz) of **4** in wet DMSO-d_6, in air, measured over one month. No changes in the ^{1}H NMR spectra were found, signifying that both the ester bond, as well as the $[(\eta^6\text{-}p\text{-cymene})Ru]^{2+}$–$[C_2B_9H_{10}]^{2-}$ fragment, are stable under these conditions.

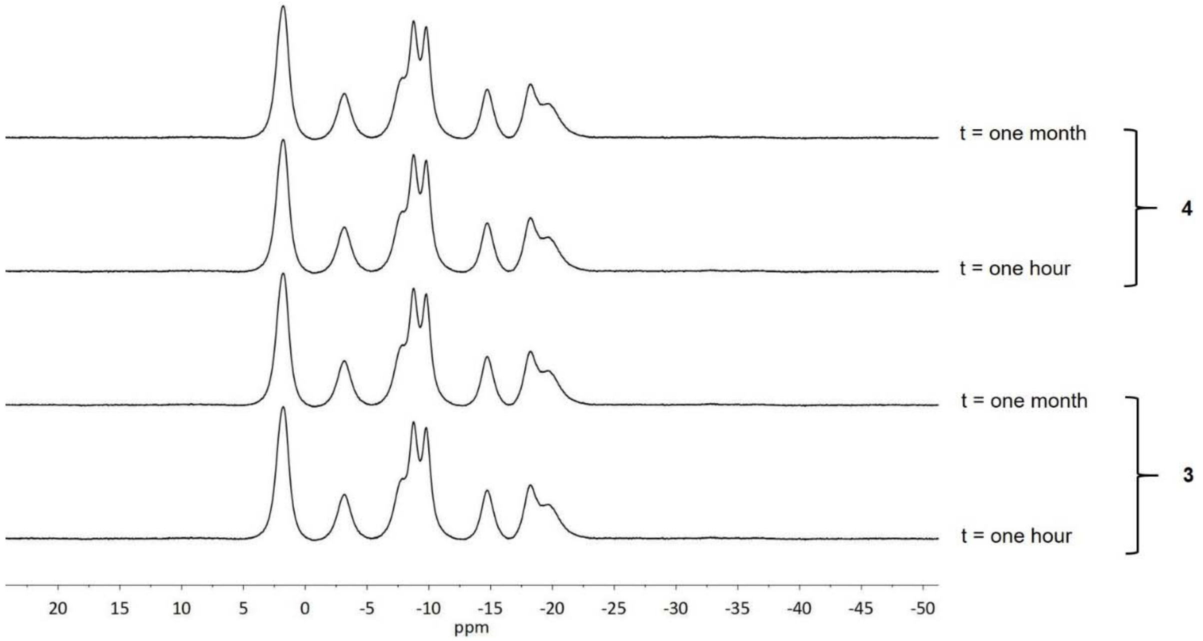

Figure S6. $^{11}B\{^{1}H\}$ NMR spectra (128.28 MHz) of **3** (top) and **4** (bottom) in wet DMSO-d_6, in air, measured over one month. No changes in the $^{11}B\{^{1}H\}$ NMR spectra were observed, signifying that the $[RuC_2B_9H_{10}]$ fragment is stable under these conditions.

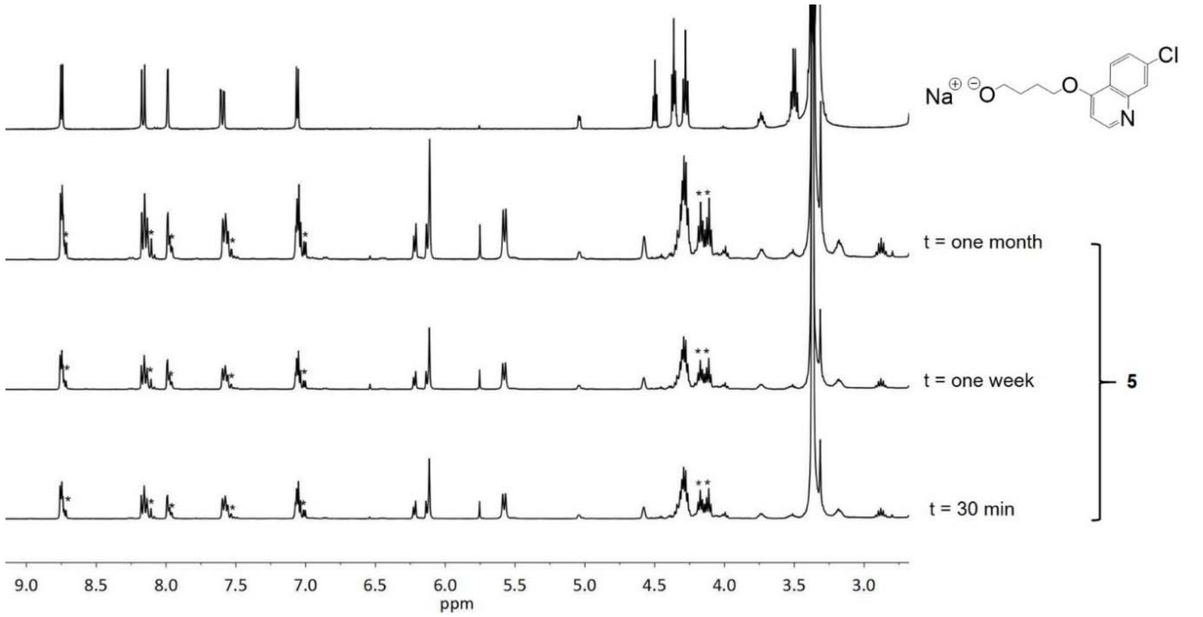

Figure S7. ^{1}H NMR spectra (400.13 MHz) of **5** in wet DMSO-d_6, in air, measured over one month. The second set of signals (second tautomer) for the {4-(7-chloroquinolin-4-yl)oxy}butanol-1-yl group are marked with *. The intensity of these signals is about 5%, with respect to the corresponding signals of **5**.

5. Crystallographic Data

5.1. Data Collection and Refinement

Table S1. Crystal data and structure refinement for **1** and **3**.

	**1**	**3**
Empirical formula	$C_4H_{14}B_{10}O_2$	$C_{14}H_{27}B_9O_2Ru$
Molecular weight	202.25 g mol^{-1}	425.71 g mol^{-1}
**Data collection**		
Reflections collected	2811	15881
Independent reflections	1945 (R_{int} = 0.0333)	3899 (R_{int} = 0.0350)
Θ_{max}	28.601°	25.242°
Completeness (%)	88.7	100.0
Crystal system	Orthorhombic	Monoclinic
Unit cell	*a* = 20.387(2) Å<br>*b* = 7.6770(7) Å<br>*c* = 6.9383(6) Å	*a* = 10.7111(2) Å<br>*b* = 13.3042(2) Å<br>*c* = 13.5954(2) Å<br>β = 100.671(2)°
Volume	1085.9(2) Å^3	1903.88(5) Å^3
Space group	$Pna2_1$	$P2_1/n$
Z	4	4
ρ_{calc}	1.237 Mg m^{-3}	1.485 Mg m^{-3}
μ(Mo-K_α)	0.068 mm^{-1}	0.828 mm^{-1}
**Refinement**		
Data/restraints/parameters	1945/1/153	3899/0/244
R (I > 2σI)	0.0540	0.0282
R_w (I > 2σI)	0.1369	0.0641
R (all data)	0.0594	0.0334
R_w (all data)	0.1412	0.0666

Max. / Min. residual electron density	0.242 / –0.183 e·Å^{-3}	0.905 / –0.358 e·Å^{-3}
Goodness-of-fit on F^2	1.077	1.063

5.2. Hydrogen Bonding in 1 and 3

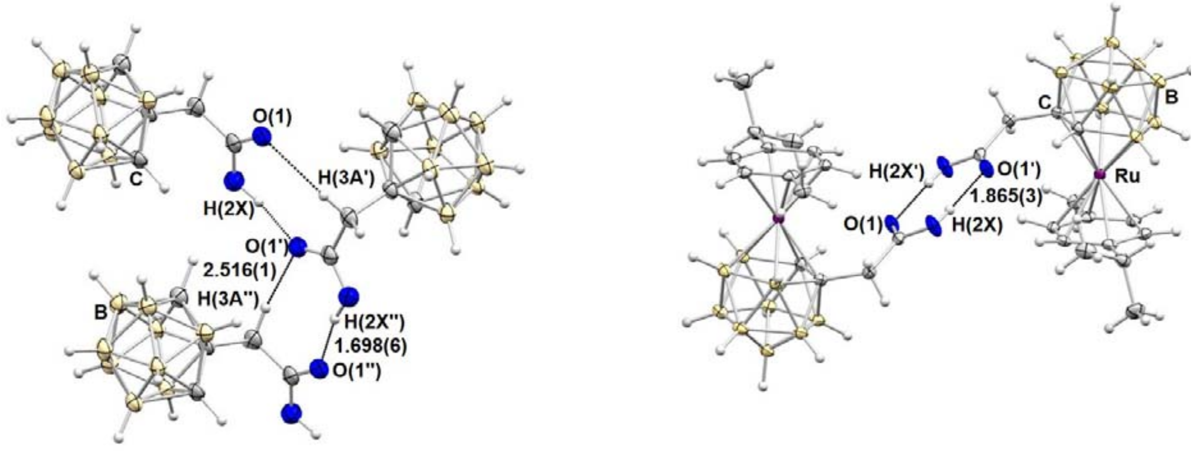

1 **3**

Figure S8. Hydrogen bonding in **1** (left) and **3** (right). Thermal ellipsoids at 50% probability level. Hydrogen bond distances are given in Å. Labelling of selected atoms is given.

6. UV-vis Spectra

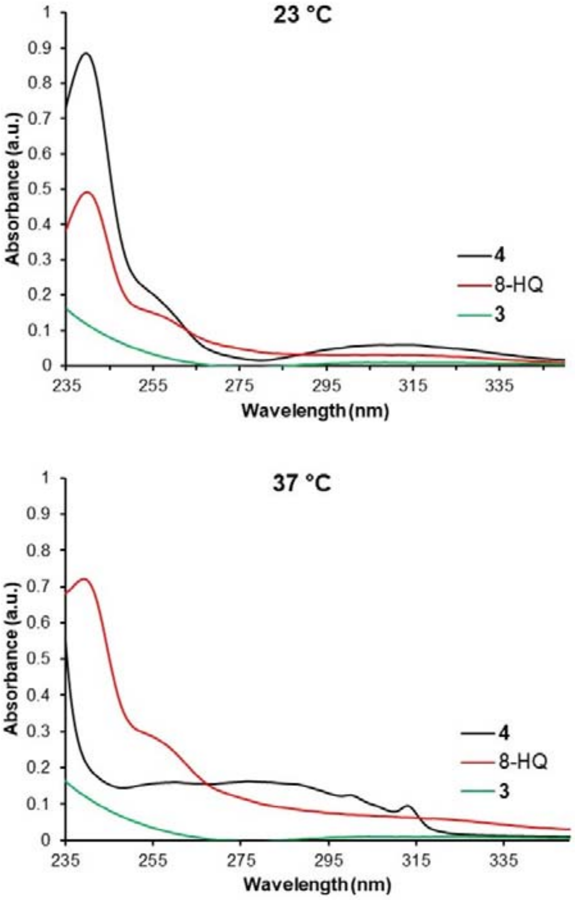

Figure S9. UV-vis spectra of **3**, **4** and 8-HQ in PBS solution, at 23 °C (top) and 37 °C (bottom), 5 min after preparation. Content of DMSO was 1 vol% in all samples. [**3**] = [**4**] = [8-HQ] = 20 µM.

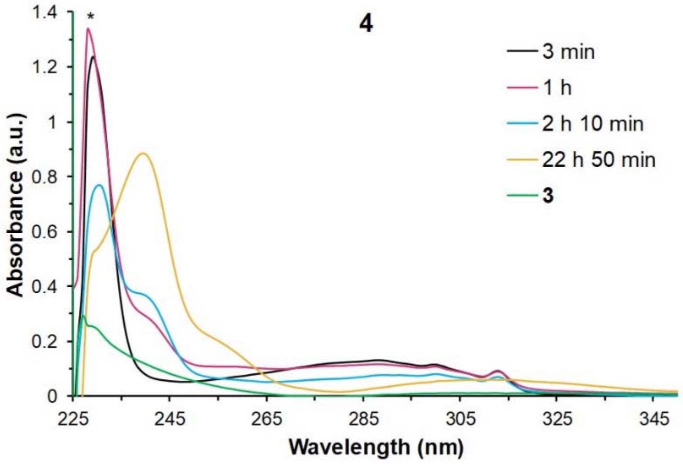

Figure S10. UV-vis spectra of **4** in PBS solution at 23 °C, at t = 3 min, 1 h, 2 h and 23 h. **3** in PBS is also shown, as reference. [**3**] = [**4**] = 20 µM. Content of DMSO was 1 vol% in all samples. * indicates the peak at λ_{max} 228–229 nm. The band at λ_{max} = 228–229 nm of **4** partially overlaps with buffer absorptions (Cl^- ions). Thus, absorption at this λ_{max} and its evolution profile are most likely the result of at least two independent phenomena, and this band was, therefore, not considered valid for following the time-resolved hydrolysis of complex **4**. This set of spectra was chosen as example. The same time-evolution in the absorbance at λ_{max} = 228–229 nm was observed at 37 °C.

7. Cell Viability

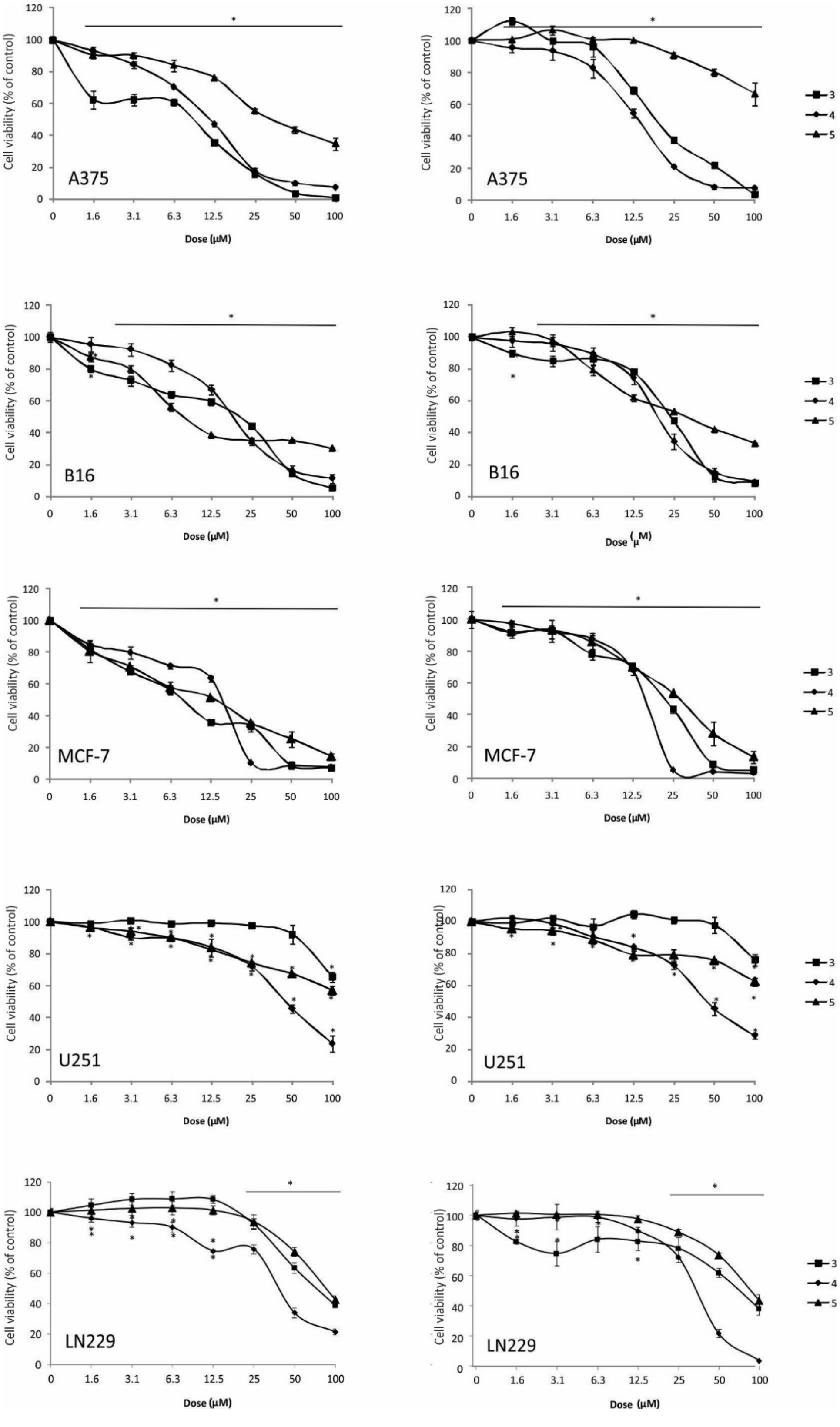

Figure S11. Cell viability curves for cancer cell lines after treatment (72 h) with **3**–**5**, from MTT (left panel) and CV (right panel) assays. Concentration of **3**–**5** varied from 1 to 100 µM. Untreated cells (control) were also measured as reference. Data are expressed as % viable cells with respect to the control, as mean value from three independent experiments. Standard deviations are also shown. * indicates statistically significant ($p < 0.05$) values, with respect to the control.

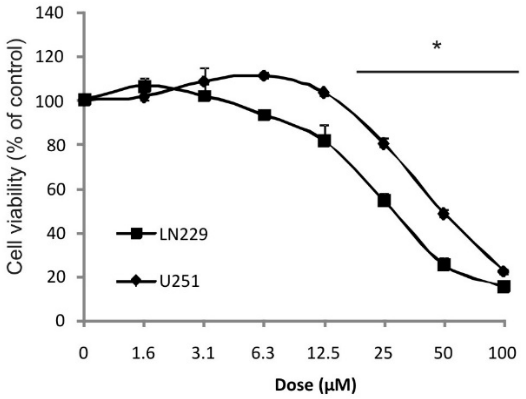

Figure S12. Cell viability curves for U251 and LN229 cell lines after treatment (72 h) with chloroquine (CQ), from CV assay. Concentration of CQ varied from 1 to 100 μM. Untreated cells (control) were also measured as reference. Data are expressed as indicated above for Figure S11. Standard deviations are also shown. * indicates statistically significant ($p < 0.05$) values, with respect to the control.

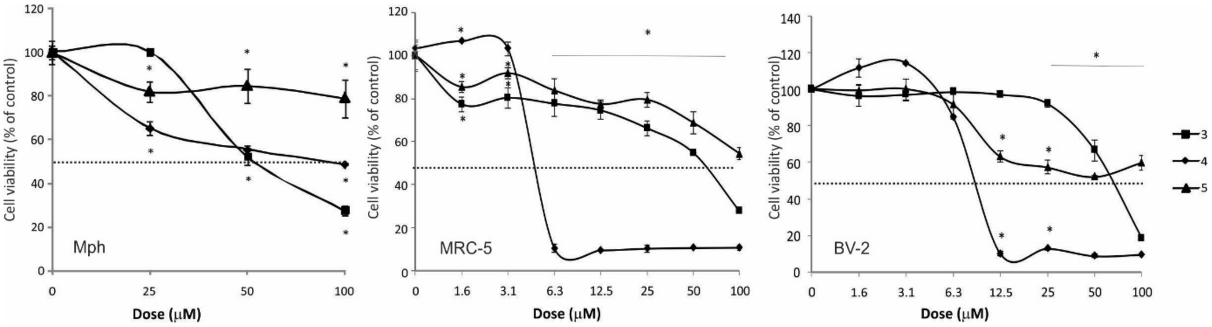

Figure S13. Cell viability curves for mouse macrophages (Mph), BV-2 and MRC-5 after treatment (72 h) with **3**–**5**, from CV assay. Concentration of **3**–**5** varied from 1 to 100 μM. Data are expressed as indicated above for Figure S11. Standard deviations are also shown. * indicates statistically significant ($p < 0.05$) values, with respect to the control. The dashed line marks the dose of **3**–**5** corresponding to 50% cell viability, with respect to the control.

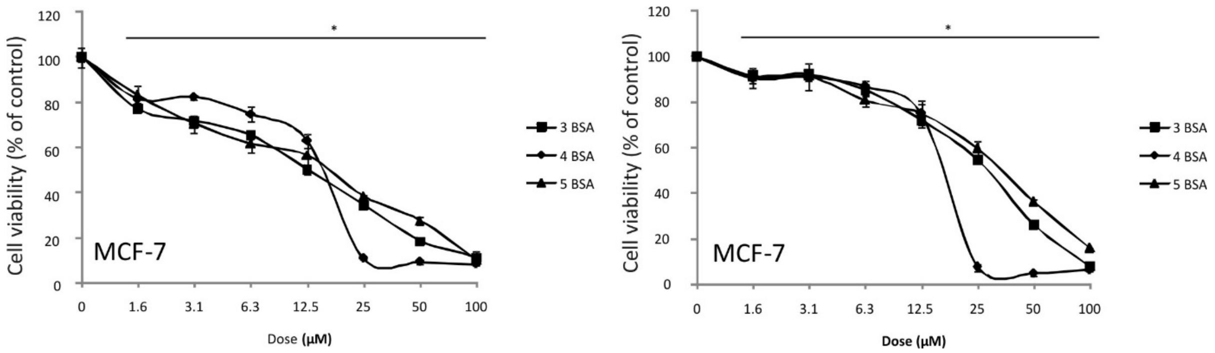

Figure S14. Cell viability curves for MCF-7 cell line after treatment (72 h) with the BSA-containing formulation of **3**–**5**, from MTT (left panel) and CV (right panel) assays. Ratio BSA:ruthenacarborane was 10:1 for all samples. Concentration of **3**–**5** varied from 1 to 100 μM. Untreated cells (control) were also measured as reference. Data are expressed as indicated above for Figure S11. * indicates statistically significant ($p < 0.05$) values, with respect to the control.

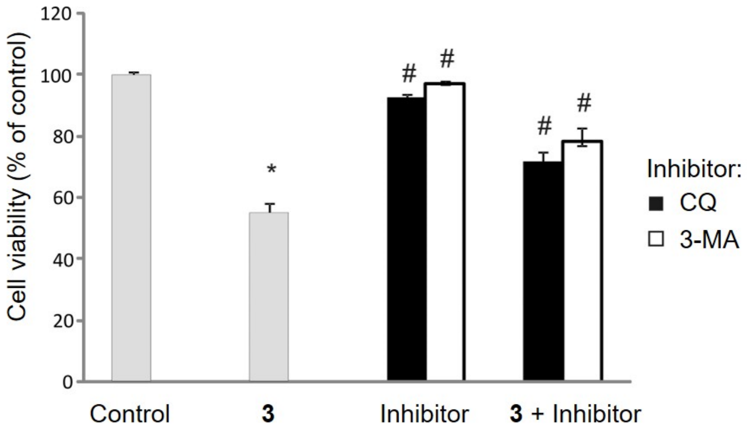

Figure S15. Cell viability for MCF-7 cell line, exposed to **3** in parallel with chloroquine (CQ, 10 µM) or 3-methyl adenine (3-MA, 0.5 mM) for 72 h, from CV assay. Data are expressed as indicated above for Figure S11. * indicates statistically significant ($p < 0.05$) values, with respect to the control, # with respect to cells treated with **3**.

8. Flow Cytometry

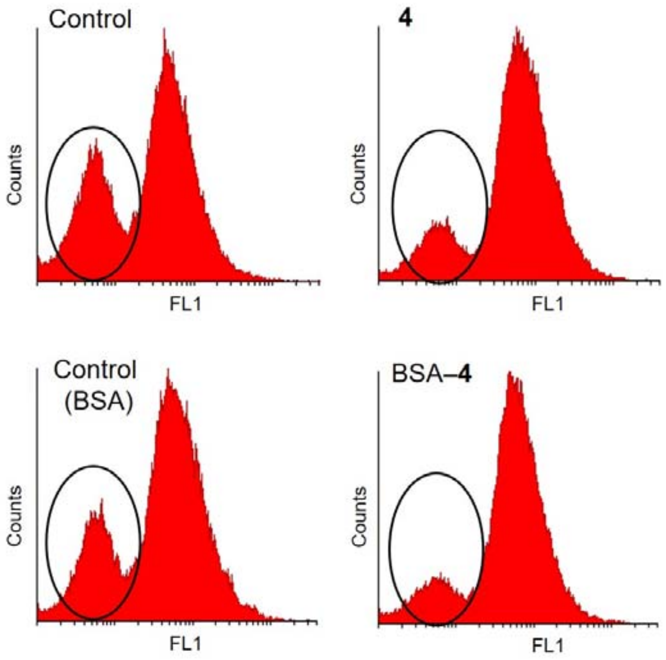

Figure S16. Detection of divided and non-divided MCF-7 cells after incubation (72 h) with **4** (top) or BSA–**4** (bottom), at 20 µM concentration of **4**. Ratio BSA:**4** was 10:1. Cells were stained with CFSE. Respective controls, i.e. untreated cells or cells treated only with BSA, were also measured for comparison. Black circles highlight the number of divided cells for each sample. (FL1, green channel)

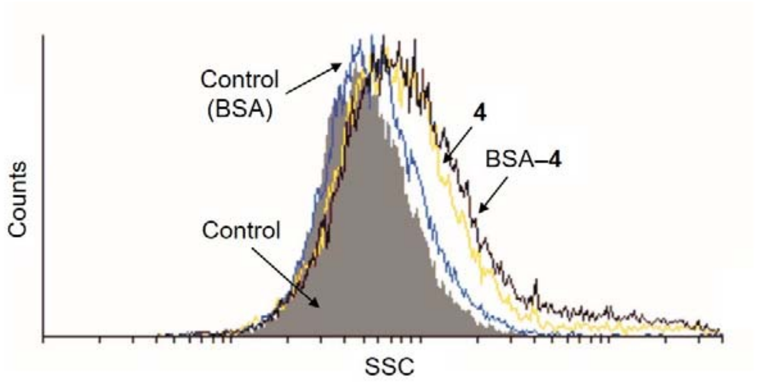

Figure S17. Sideward Scatter (SSC) plot of MCF-7 cells incubated (72 h) with **4** or BSA–**4**, at 20 µM concentration of **4**. Ratio BSA:**4** was 10:1. Respective controls, i.e. untreated cells or cells treated only with BSA, were also measured for comparison. Sideward Scatter is proportional to cell granularity and was determined via flow cytometry.

9. List of Abbreviations

3-MA	3-methyladenine
8-HQ	8-hydroxyquinoline
Ann(V)	annexin V
AO	acridine orange
BBB	blood-brain barrier
BNCT	boron neutron capture therapy
BSA	bovine serum albumin
CFSE	carboxyfluoresceinsuccinimidyl ester
CNS	central nervous system
CQ	chloroquine
CV	crystal violet
DAPI	4′,6-diamidino-2-phenylindole
DMEM	Dulbecco's Modified Eagle Medium
DMF	dimethylformamide
DMSO	dimethylsulfoxide
EPR	enhanced permeability and retention
FCS	fetal calf serum
GBM	glioblastoma
HCQ	hydroxychloroquine
HEPES	4-(2-hydroxyethyl)-1-piperazineethanesulfonic acid
LC3B	microtubule-associated protein light chain 3B
LMCT	ligand-to-metal charge transfer
MLCT	metal-to-ligand charge transfer
MTT	3-(4,5-dimethylthiazol-2-yl)-2,5-diphenyltetrazolium bromide
NTA	Nanoparticle Tracking Analysis

PBS	phosphate-buffered saline
PI	propidium iodide
SDS	sodium dodecylsulfate
TLC	thin-layer chromatography
THF	tetrahydrofuran
TMZ	temozolomide
